# Rice-eel system combined with exogenous organic waste improves soil quality under nitrogen deficiency by regulating soil microbial community

**DOI:** 10.3389/fmicb.2025.1743071

**Published:** 2026-01-14

**Authors:** Yanan Pei, Zihan Yuan, Weiguang Lv, Siyu Qiu, Chenglong Xu, Xianpu Zhu, Shuangxi Li, Juanqin Zhang, Naling Bai, Haiyun Zhang, Hanlin Zhang

**Affiliations:** 1 Eco-Environmental Protection Institute, Shanghai Academy of Agricultural Sciences, Shanghai, China; 2 Key Laboratory of Low-Carbon Green Agriculture in Southeastern China, Ministry of Agriculture and Rural Affairs, Shanghai, China; 3 National Agricultural Experimental Station for Agricultural Environment, Shanghai, China; 4 Key Laboratory of Integrated Rice Fish Farming Ecosystem, Ministry of Agriculture and Rural Affairs, Shanghai, China

**Keywords:** microbial community, organic fertilizer substitution, rice-eel system, sustainable agriculture, water stability of aggregates

## Abstract

**Background:**

The rice-eel system, a cultivation method integrating aquatic animals with rice, offers ecological and agronomic advantages, yet its long-term effects of substituting chemical fertilizers with straw or organic fertilizers on soil properties remain unclear.

**Aims:**

This study aimed to quantify the effects of organic waste substitutions within the rice-eel system on soil physicochemical properties and microbial communities.

**Methods:**

A 2016-established field experiment on sandy loam soil under rice-fallow rotation, and soil samples (0–20 cm and 20–40 cm depths) were taken in March 2025. The study included five treatments: conventional fertilization (100%) without eel (RT), conventional fertilization (100%) + eel (IRT), 70% chemical fertilization + eel (I70), 70% chemical fertilization + 30% straw + eel (IS), and 70% chemical fertilization + 30% organic fertilizer + eel (IO).

**Results:**

The system improved soil macroaggregate stability, with the strongest effects under IS and IO. Compared with RT, IS and IO significantly increased soil organic matter (SOM) by 16.04% on average, at 0–20 cm, and increased SOM and available phosphorus (AP) by 18.60 and 33.70%, respectively, at 20–40 cm. IS and IO significantly increased bacterial and fungal gene copies by an average of 64.95% (0–20 cm) and 76.20% (20–40 cm). The rice-eel system improved microbial diversity, reshaped community composition, and increased taxa such as Chloroflexi, Acidobacteriota, Pleosporales and Chytridiomycota which contribute to organic matter decomposition and aggregate formation. The relative abundance of microorganisms associated with aerobic respiration (cytochrome c pathway) increased, while functional pathways related to biosynthesis and degradation/utilization/assimilation were also strengthened.

**Conclusion:**

The rice-eel system—particularly IO—significantly improved fertility, aggregate stability, and microbial function. These findings indicate that the rice-eel system reduces reliance on chemical fertilizers while sustaining productivity, offering a practical strategy for ecological agriculture.

## 1 Introduction

Co-cultivation of rice and aquatic animals is a prominent agricultural practice in the rice-growing regions of southern China. Based on principles of ecological symbiosis and functional complementarity, this system optimizes spatial and resource utilization within shallow-water paddy environments (Xu et al., 2022). Integrating aquatic species such as fish, crabs, or crayfish into rice cultivation has been widely shown to enhance soil biological activity and economic returns, primarily through improved nutrient availability, reduced pest pressure, and more efficient field management, thereby increasing rice yield and profitability (Hou et al., 2021; Xie et al., 2011). Previous studies on rice-fish, rice-shrimp, and rice–crayfish systems have consistently demonstrated these agronomic and economic benefits. However, most existing research has been based on short-term experiments and has focused predominantly on yield-related traits and surface-level soil nutrient responses. Such approaches are insufficient to capture the cumulative and time-dependent processes governing soil structural development, nutrient stabilization, and microbial community succession under integrated planting-breeding systems. In particular, soil aggregate formation, organic carbon sequestration, phosphorus transformation, and microbial network reorganization typically occur over multi-year timescales and cannot be reliably assessed through short-term observations. In addition to economic benefits, this integrated system contributes to environmental sustainability by reducing chemical fertilizer inputs, enhancing in-field water purification, and mitigating non-point source pollution (Hou et al., 2024; Kaewpuangdee et al., 2024). Systems such as rice-river crab and rice-crayfish can reduce methane emissions by up to 22%, highlighting their potential role in climate change mitigation (Cui et al., 2023; Yu et al., 2023). Moreover, these systems help prevent land degradation and maintain agroecosystem stability by coupling biological disturbance with nutrient recycling (Zhang et al., 2023). Correspondingly, long-term field evidence indicates that rice-aquatic animal systems enhance soil macroscopic aggregate formation, increase soil organic carbon (SOC) stocks, and improve phosphorus availability, thereby contributing to sustained soil quality improvement (Lin and Wu, 2020; Si et al., 2017; Zhang et al., 2022). Consequently, promoting ecological farming and breeding models in rice fields is crucial for the sustainable development of agriculture.

Soil microorganisms are crucial indicators of soil quality and health, playing essential roles in soil fertility and nutrient cycling. The rice-aquatic animal system has been shown to alter the structure of soil microbial communities. Si et al. (2017) reported that the rice-shrimp system enhanced both soil microbial activity and diversity. Similarly, Wu et al. (2024) found that the rice-snail-crayfish system significantly modified the microbial community composition, increasing the abundance of bacterial phospholipid fatty acids, Gram-negative bacteria, anaerobic bacteria, and arbuscular mycorrhizal fungi. Nevertheless, microbial communities respond not only to management practices but also to prolonged changes in soil physical structure, nutrient pools, and microbial habitats driven by eel-induced bioturbation, organic matter inputs, and fertilization strategies—processes that accumulate gradually over time rather than responding immediately and therefore require long-term field observation to be fully understood. Compared with rice–fish and rice–shrimp systems, long-term studies on rice–eel co-cultivation remain particularly limited, especially with respect to soil physical structure, nutrient cycling processes, and microbial community dynamics. Eels thrive in shallow water environments, such as ditches and rice fields, and adapt well to environmental conditions. During the night, they leave the ditches and burrow into the paddy fields, creating mud holes suitable for their habitat (Aquino et al., 2021; Edeline et al., 2005). This continuous bioturbation can progressively modify soil porosity, aeration status, and microscale habitat heterogeneity, effects that are expected to intensify over time rather than manifest immediately (Correia-da-Silva et al., 2017; Tomie et al., 2013). Compared to common fish, eels possess both high economic value and certain medicinal properties. In addition, eel excreta and residual feed inputs contribute organic matter to the soil, promoting nutrient cycling, reducing external nitrogen dependence, and enhancing rice productivity through cumulative effects on soil fertility (Fan et al., 2024; Lv et al., 2020). Meanwhile, the rice-eel system shows remarkable performance in reducing antibiotic use and managing antibiotic resistance genes, and it could also affect weed management (Zhao et al., 2025). In this framework, existing studies on the rice-eel system predominantly span 1–4 years and primarily concentrate on aspects including soil nutrient levels, antibiotic resistance genes, bacterial community composition, water quality, and the biomass of weeds and pests. There are still relatively few studies on the long-term effects on soil microbial communities, soil physical structure and chemical properties.

The substitution of chemical fertilizers with straw and organic fertilizers is a widely adopted ecological agricultural strategy. Returning straw to the field and applying organic fertilizers can optimize soil structure, promote nutrient cycling, and reduce reliance on chemical fertilizers (Xue et al., 2024; Yang et al., 2025; Yuan et al., 2025). Previous studies on integrated planting and breeding models have shown positive results, but few have explored the coupled application of the rice-eel system with straw returning to the field and organic fertilizer application. This study used a long-term ecological rice-eel system established in 2016 to examine the effects of organic fertilizer and straw substitution on soil structure, nutrient dynamics, and microbial community composition. Specifically, the objectives of the study were to: (1) determine how substituting chemical fertilizers with organic matter affects soil aggregate stability and nutrient dynamics in the rice-eel system; (2) elucidate the composition, functional potential, and spatial distribution of bacterial and fungal communities under rice-eel system; and (3) determine how specific microbial taxa mediate soil quality improvements through the dynamic interactions among nutrients, soil structure, and microbial processes under the rice-eel system with organic fertilizer substitution. The findings are expected to provide a scientific basis for optimizing and promoting the rice-eel system as a sustainable cultivation practice that enhances soil fertility, structural stability, and microbial functionality while reducing chemical fertilizer dependency.

## 2 Materials and methods

### 2.1 Site description

The experiment began in May 2016 during the rice-growing season. Annually, consistent practices including rice-fallow rotation, field management, and fertilization strategies were implemented. The long-term rice-eel system experiment was conducted on sandy loam soil at the Zhuanghang Comprehensive Experiment Station of the Shanghai Academy of Agricultural Sciences (30 °53′N, 121 °23′E). The site is characterized by a subtropical monsoon climate, with a mean annual rainfall of 1,241.1 mm and an average temperature of 16.5 °C. The rice cultivar used was Huayou 14 (*Oryza sativa* L.), and the eel species was dark-spotted yellow eel (*Monopterus albus*). Soil samples were collected in March 2025 for subsequent analyses.

### 2.2 Experimental design

**Figure 1:**
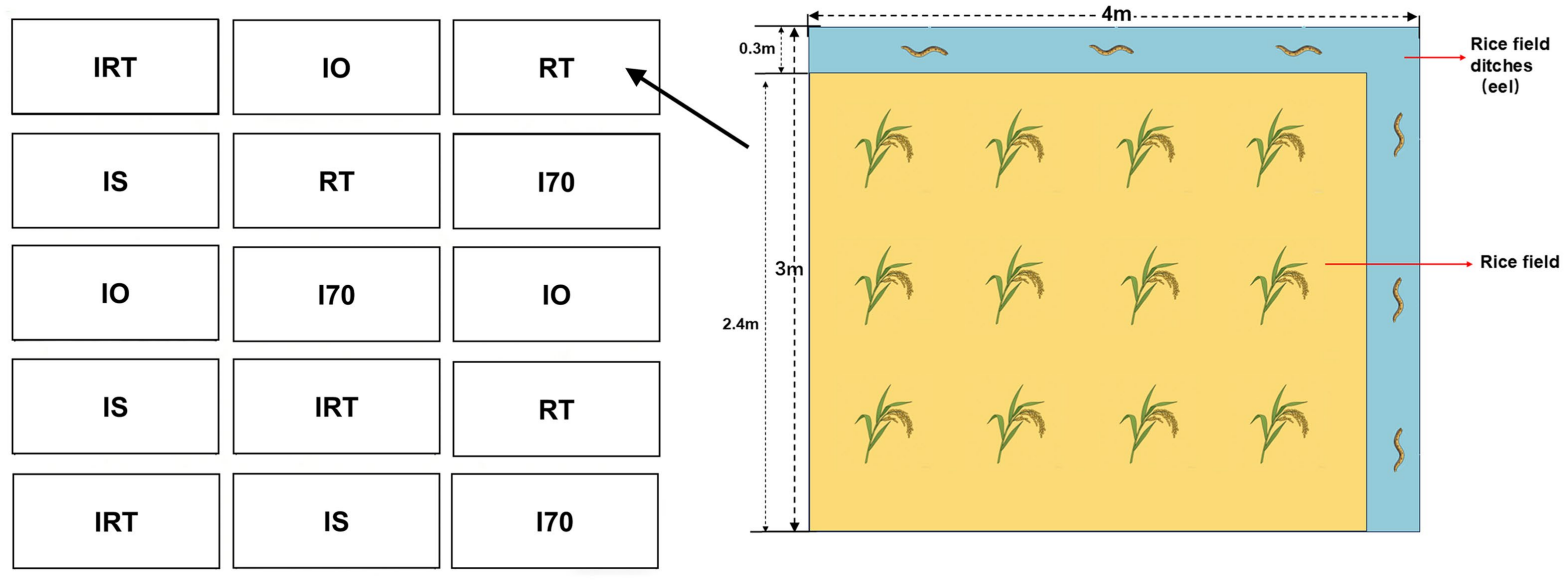
The rice-eel co-culture system consisted of a rice field surrounded on two sides by a ditch. On the left is the layout of 15 experimental fields, and on the right are the details of the experimental fields.

Five treatments were established using a randomized block design (Table 1), and each replicated three times, resulting in a total of 15 plots, each measuring 4 m × 3 m. Each plot contained an L-shaped trench occupying 15% of the plot area (Figure 1). The plots were enclosed with a geomembrane to ensure the isolation of fertilizer and water. Eels were released into the ditch in the paddy fields during the seedling stage of rice. The fry had body weight of 20–25 g and a length of 27–31 cm, and the put density was 100 kg·ha^−1^. The eels relied on natural feed sources such as earthworms, insects, and aquatic plankton, no supplemental feed was provided.

**Table 1:** Fertilization regime of different treatments.

Treatment	Whether to breed eel	Type and dosage of fertilizers (pure nitrogen season)
RT	No	100% chemical fertilizer
IRT	Yes	100% chemical fertilizer
I70	Yes	70% chemical fertilizer
IS	Yes	70% chemical fertilizer + 30% straw
IO	Yes	70% chemical fertilizer + 30% organic fertilizer

Fertilization followed local agricultural practices, RT and IRT applying 300 kg·ha^−1^ of pure nitrogen (N), 192 kg·ha^−1^ of phosphorus (P₂O₅), and 180 kg·ha^−1^ of potassium (K₂O) during the rice season. The fertilizers used included urea, superphosphate, and potassium sulfate. In the treatments of I70, IO, and IS, applied 70% of total pure nitrogen in chemical fertilizers. In IO and IS, the remaining 30% of pure nitrogen was substituted with organic fertilizer and straw, respectively. The organic fertilizer, provided by Shanghai Ouhai Energy Technology Co., Ltd., was composed primarily of fermented rice straw material and contained 2.51% N (N), 1.02% P (P₂O₅), and 1.53% K (K₂O). The straw contained 0.33% N, 0.19% P, and 1.35% K. After the rice harvest, straw was crushed and deeply plowed into the soil to a depth of approximately 20 cm. Organic fertilizer was applied in its entirety as a basal dressing for rice in a single application. The nutrient contents of the organic fertilizer and straw were included in the total fertilizer calculations based on their measured values. If phosphorus, and potassium levels were insufficient, additional chemical fertilizers were applied to ensure nutrient consistency across all treatments.

### 2.3 Soil sampling

Soil samples were collected by soil samplers in March 2025 using the five-point sampling method from two soil depths: 0–20 cm and 20–40 cm. For each depth, five samples were taken within a plot and mixed thoroughly to form one, which had considered one replicate. A total of 3 replicates were collected for each treatment. Immediately after collecting, visible plant residues, roots, and gravel were manually removed, and placed into plastic self-sealing bags for transportation to the laboratory within 24 h. Upon arrival at the laboratory, a portion of fresh soil was immediately frozen in liquid nitrogen and stored at −80 °C until DNA extraction for high-throughput sequencing and quantitative PCR (qPCR) analyses, to preserve microbial community structure and nucleic acid integrity. The remaining soil was air-dried at room temperature and subsequently divided into two portions. One portion was gently ground and sieved through 10 mesh (≥2 mm) and 100 mesh (<2 mm) sieves to obtain coarse and fine fractions for physicochemical analyses. The two soil fractions were then separately placed into sterile self-sealing bags. Another portion was used for the determination of soil aggregate characteristics. The other portion was used for the determination of soil aggregate characteristics.

The wet-sieving method described by Elliott (1986) was used to determine the distribution of soil aggregates across different particle size classes. First, air-dried soil clods were broken along their natural structural surfaces into smaller clods of 1-2 cm in diameter. Visible plant residues and stones were removed, and 50 g of the processed sample was weighed for analysis. The soil was pre-moistened with water and evenly distributed on the top sieve (2 mm) of a set of nested sieves with mesh sizes of 2, 1, 0.5, 0.25, and 0.053 mm. The sieve stack was placed into a soil aggregate analyzer filled with water, ensuring the sieves were fully moistened. Wet-sieving was performed with an amplitude of 3.8 cm and a vibration frequency of 30 oscillations per minute for 30 min. After sieving, the aggregates retained on each sieve were rinsed into separate beakers with distilled water, oven-dried, and weighed. The soil was fractionated into six particle size classes: >2 mm, 1-2 mm, 0.5–1 mm, 0.25–0.5 mm, 0.053–0.25 mm, and <0.053 mm. The proportion of aggregates in each size class and their water stability were calculated.

Equations (1)–(3) show the calculation formulas for the number of water-stable large aggregates (R0.25), mean weight diameter (MWD), and geometric mean diameter (GMD).”


$$R_{0.25}=\frac{M_{r>0.25}}{M_{T}}$$ (1)

$$\mathrm{MWD}=\sum_{i=1}^{n}X_{i}W_{i}$$ (2)

$$\mathrm{GMD}=\exp(\frac{\sum_{i=1}^{n}W_{i}\ln X_{i}}{\sum_{i}^{n}W_{i}})$$ (3)

The formula is explained as follows: $M_{r>0.25}$ represents the mass (g) of aggregates with a particle size greater than 0.25 mm; $M_{T}$ is the total weight (g) of the aggregate; $X_{i}$ is the average diameter (mm) of aggregates in the *i*-th grain size class; $W_{i}$ represents the percentage content (%) of aggregates of the *i*-th grain size class in the total aggregates.

### 2.4 Soil chemical properties and crop yield analysis

Soil Total nitrogen (TN) was determined using the sulfuric acid digestion method (Bremner, 1960). Both total phosphorus (TP) was measured via sulfuric acid-perchloric acid digestion. Available nitrogen (AN) was assessed using the Kjeldahl digestion method. Available phosphorus (AP) was measured using the sodium bicarbonate extraction-molybdate scandium colorimetric method. Soil organic matter (SOM) content was analyzed using the potassium dichromate-sulfuric acid oxidation method, and the pH was determined by the soil potential method (water and soil ratio 2.5:1) (Bao, 2000).

### 2.5 Microbial analysis

A 0.5 g soil sample was used for DNA extraction with the PowerSoil^®^ DNA Isolation Kit (MoBio Laboratories, United States), following the manufacturer’s protocol.

Quantitative PCR analysis: Bacterial and fungal communities were amplified using the primer pairs 341F/806R and ITS1F/ITS2R, respectively. Quantitative PCR (qPCR) was performed in a 30 μL reaction volume containing 15 μL of qPCR Mix, 2 μL of Mg^2+^ (25 mM), 0.5 μL each of forward and reverse primers, 2 μL of DNA template, 0.5 μL of fluorescent dye, and nuclease-free water to a final volume of 30 μL. The thermal cycling conditions were initial denaturation at 95 °C for 3 min, followed by 34 cycles of bacteria and 35 cycles of fungi, respectively. Each cycle consisted of denaturation at 94 °C for 30 s, annealing at 50 °C (bacteria) and 59 °C (fungi) for 30 s, and extension at 72 °C (bacteria) and 81 °C (fungi) for 30 s, respectively; followed by a final extension at 72 °C for 10 min.

High-throughput analysis: The community structure of soil bacteria in paddy fields was investigated by sequencing the V3-V4 region of the 16S rRNA gene. Amplification was performed using primers 338F (5′-ACTCCTACGGGAGGCAGCA-3′) and 806R (5′-GGACTACHVGGGTWTCTAAT-3′) as described by Bergmann et al. (2011). The fungal internal transcribed spacer 1 (ITS1) region was amplified using the fungus-specific primers ITS1F (5′-CTTGGTCATTTAGAGGAAGTAA-3′) and ITS2 (5′-GCTGCGTTCTTCATCGATGC-3′), following the protocol of Schoch et al. (2012). PCR conditions were: initial denaturation at 94 °C for 2 min, followed by 35 cycles for bacteria and 45 cycles for fungi. Each cycle consisted of denaturation at 94 °C for 30 s, annealing at 50 °C for 30 s, and extension at 72 °C for 30 s. After purification and quantification of the PCR products, high-throughput sequencing was performed. The samples were submitted to Shanghai Personal Biotechnology Co., Ltd. for sequencing analysis using the Mesiq platform, sequences (similarity more than 97%) were clustered into Operational Taxonomic Units (OTUs), which were subsequently annotated to yield taxonomic information.

### 2.6 Statistical analysis

A one-way analysis of variance (ANOVA) followed by Tukey’s *post hoc* test (*p* < 0.05) was performed to analyze 240 samples across 5 treatments and 6 nutrient indices (TN, TP, AN, AP, SOM, pH) using SPSS 26.0. PERMANOVA analysis was conducted using SPSS 26.0. Nutrient indices, Correlation analysis were visualized with Origin 9.0. Redundancy Analysis (RDA), Microbial analysis and visualization were conducted on the Personalbio Genescloud Platform.

Grey relational analysis (GRA) quantifies the influence of factors within an index system to identify optimal treatments (Liu et al., 2011). Widely applied in economics, marketing, medicine, computer science, and social sciences, GRA supports robust decision-making (Li et al., 2013; Lv and Du, 2012). In this study, we applied GRA to evaluate the optimal fertilization method of the rice-eel system. We constructed the grey system using 10 indexes and 5 treatments, encompassing a total of 50 samples. We then calculated the Grey Relational Degree for each treatment, with higher values indicating more effective soil improvement. The calculation process of grey relational degree analysis is as follows (Cheng and Ma, 2011).

Given the large differences in the evaluation indexes, it is essential to conduct standardized. According to Equation 1, the original data are transformed, Standardized data $y_{k}$ is given by:


$$y_{k}=\frac{X_{i}}{X_{a}}$$ (4)

In Equation 4, $X_{i}$ represents the original data, and $X_{a}$ represents the average value of the *k*-th index of *m* samples.

Since the magnitude and unit of each target reflection are different, it is necessary to make them dimensionless. The dimensionless value ωi are shown in Supplementary Table S1 and is given by:


$$\mathrm{\omega i}=\frac{y_{\max}-y_{k}}{y_{\max}-y_{\min}}$$ (5)

In Equation 5, $y_{\max}$ , $y_{\min}$ represents the maximum and minimum value of $y_{k}$ , respectively.

Using the dimensionless data obtained by calculation, the grey relation coefficient $\varepsilon_{i}$ of *i* sequence is calculated and shown in Supplementary Table S2.


$$\varepsilon_{i}=\frac{|a-y_{\min}|+\rho |a-y_{\max}|}{|a-y_{d}|+\rho |a-y_{\max}|}\;$$ (6)

In Equation 6, *ρ* is the resolution coefficient, it’s usually 0.5 and a usually 0.

Grey Relation Degree $\beta_{i}$ is shown in Equation (7)


$$\beta_{i}=\frac{1}{n}\sum_{i=1}^{n}\varepsilon_{i}$$ (7)

## 3 Results

### 3.1 Soil water-stable aggregates

**Figure 2:**
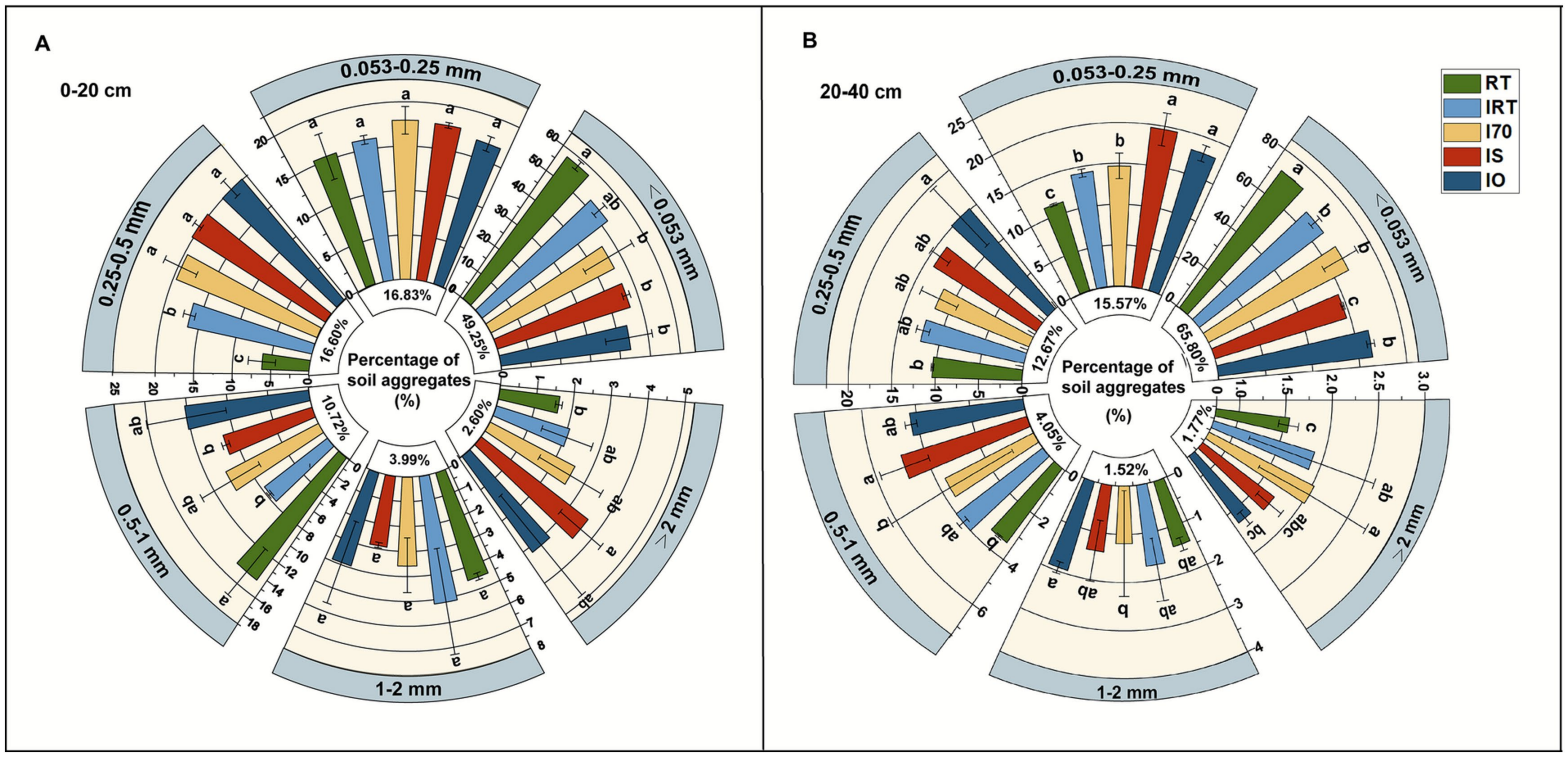
The particle size distribution of soil aggregates at 0–20 cm **(A)** and 20–40 cm **(B)** under different treatments. Different low case letters above columns indicate statistical differences at *p* < 0.05.

Figure 2 illustrated the distribution of soil water-stable aggregates under various treatments. In the 0–20 cm soil (Figure 2A), the largest proportion of particle size was <0.053 mm, averaging 49.25%. Compared to RT, the rice-eel system showed a significantly higher content of 0.25–0.5 mm aggregates, with an average increase of 208.94%. In addition, IS also significantly increased the proportion of >2 mm aggregates by 121.62% and reduced those sized 0.5–1 mm by 37.56%. In contrast, compared to RT <0.053 mm aggregates were significantly reduced in the rice-eel system (excluding IRT), with an average reduction of 21.36%. In the 20–40 cm soil (Figure 2B), the largest proportion of particle size still was <0.053 mm, averaging 65.80%, which was higher than that of 0–20 cm. Compared to RT, the proportion of >2 mm aggregates increased significantly under IRT and I70 by 16.34 and 24.37%, respectively. The proportion of 0.5–1 mm aggregates under IS and 0.25–0.5 mm aggregates under IO also increased significantly by 42.44 and 46.05%, respectively. Additionally, compared to RT, 0.053–0.25 mm aggregates in the rice-eel system were increased by an average of 46.68%, while those <0.053 mm aggregates decreased by an average of 10.61%.

**Figure 3:**
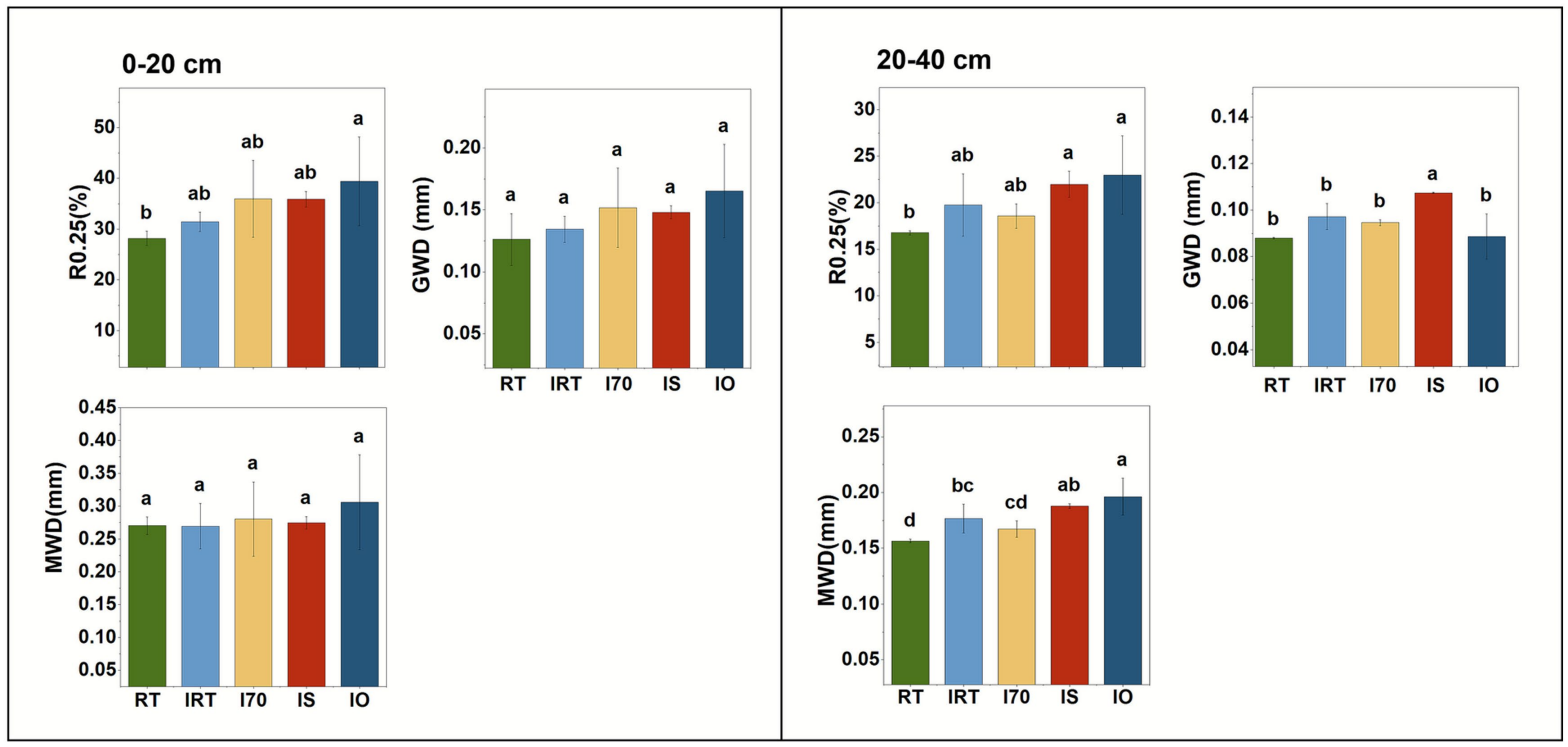
Effects of rice-eel system on water stability of soil aggregates at 0–20 cm and 20–40 cm. Different low case letters above columns indicate statistical differences at *p* < 0.05.

The water-stable aggregates under different fertilization treatments were shown in Figure 3. Compared with RT, in both the 0–20 cm and 20–40 cm soil, the rice-eel system generally increased soil R0.25, GMD and MWD numerically. Notably, in 0–20 cm, only IO significantly increased soil R0.25, which was 39.88% higher than RT. There was no significant difference in GMD and MWD among all the treatments. In 20–40 cm, compared to RT, R0.25 and MWD were significantly higher under IS and IO, with average increases of 34.07 and 22.63%, respectively. Additionally, GWD under IS was significantly higher than RT by 22.19%.

### 3.2 Soil chemical properties

**Figure 4:**
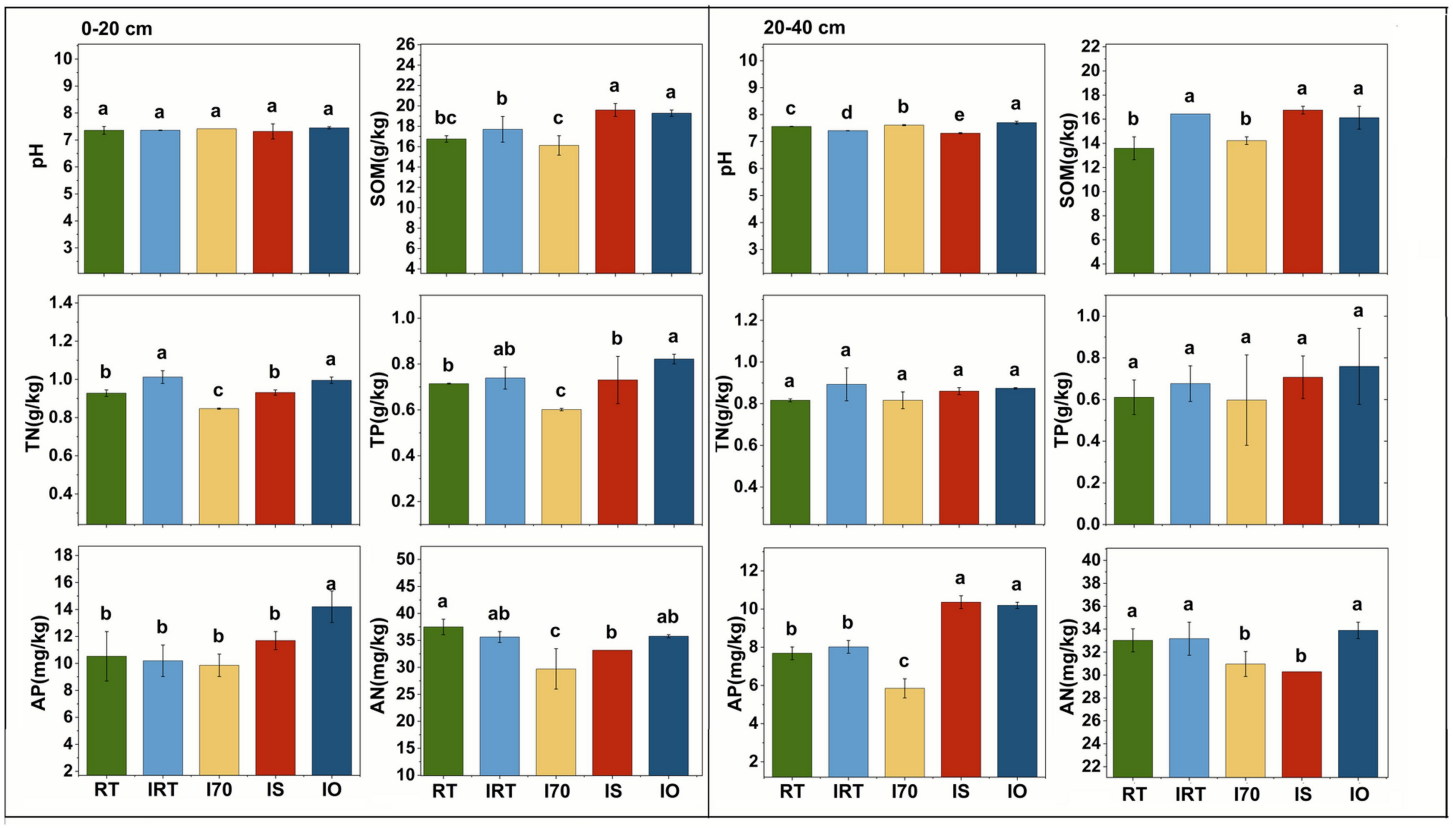
Effects of rice-eel system on soil nutrients at 0–20 cm and 20–40 cm under different treatments. The left side of each figure is 0–20 cm, and the right side is 20–40 cm. Different low case letters above columns indicate statistical differences at *p* < 0.05.

The effects of the rice-eel system and different fertilization methods on soil chemical properties were shown in Figure 4. In the 0–20 cm soil, there was no significant difference in pH among all the treatments, with an average value of 7.38. Compared with RT, IS and IO significantly increased SOM by 16.04% on average. TN under IRT and IO were significantly higher than those of RT by 8.18% on average. AN under I70 and IS were 16.15% on average lower than that under RT. IO had the highest TP and AP, which was significantly higher than RT by 15.05 and 34.92%, respectively. SOM reached peaks in IS, which was significantly 16.98% higher than those of RT. In contrast, I70 showed the lowest values for SOM, TN, TP, and AN, which were significantly 3.77, 8.73, 15.86, and 20.77% lower than those of RT, respectively.

In 20–40 cm soil, nutrient content was generally lower than that in the 0–20 cm soil (Figure 4). The average pH increased significantly to 7.66 under I70 and IO, while IRT and IS decreased significantly by an average of 2.68%. The rice-eel system had no significant effects on TN and TP. Compared with RT, SOM significantly increased by an average of 20.93% in IRT, IS and IO. Compared with RT, AN under I70 and IS was significantly lower by 6.26 and 8.30% respectively, while the average of AP under IS and IO was significantly higher than RT by 33.70%. Among all the treatments, IS achieved the peak of SOM and AP, which were significantly higher than RT by 23.26 and 34.78% respectively, while I70 had the lowest AP, which was significantly lower than RT by 23.91%.

### 3.3 Soil microbial community abundance and diversity

**Figure 5:**
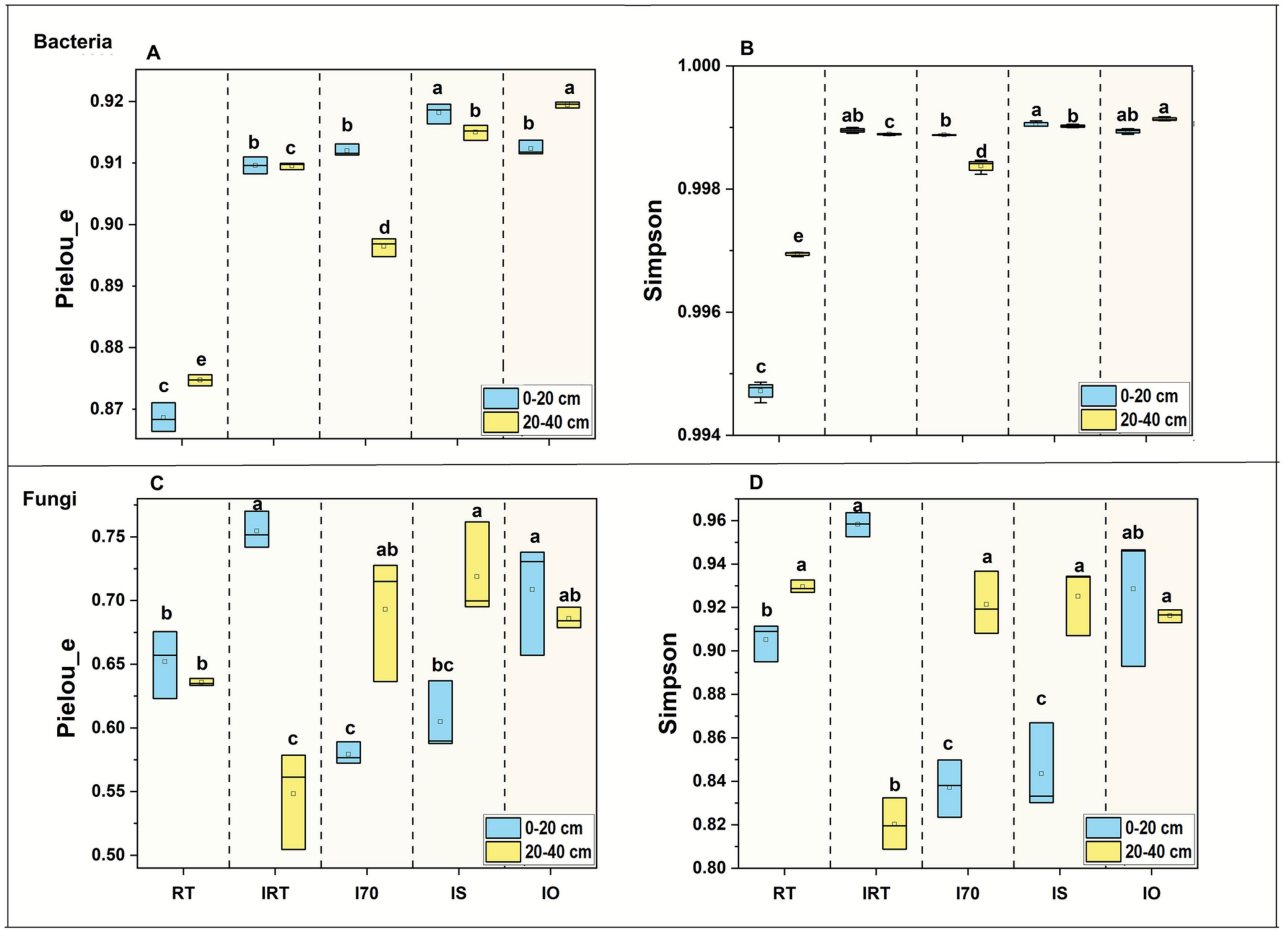
The effects of rice-eel system on bacterial and fungal diversity at different soil layers. The bacterial and fungal diversity is shown in **(A–D)**, respectively. Different low case letters above columns indicate statistical differences at *p* < 0.05.

In the 0–20 cm soil, compared with RT, the rice-eel system significantly increased bacterial community Pielou_e and Simpson by an average of 5.11 and 0.43%, respectively, reaching their highest values under IS-0.44 and 8.31% higher than RT (Figure 5A). At 20–40 cm, compared with RT, the rice-eel system significantly enhanced Simpson and Pielou_e of the bacterial community, increasing by an average of 0.19 and 4.05%, respectively (Figure 5B). Compared to RT, IO achieved peak bacterial community diversity, which was significantly increased by 0.22% (Simpson) and 2.48% (Pielou_e).

At 0–20 cm, compared to RT, IRT significantly increased the Simpson of fungal community by 5.87%, while I70 and IS significantly decreased Simpson by 7.52 and 6.82%, respectively (Figure 5C). For Pielou_e, IRT and IO showed significant increases of 15.73 and 8.68%, respectively, whereas I70 caused a significant decrease of 11.14%. At 20–40 cm, compared to RT, IRT significantly reduced Simpson diversity and Pielou_e by 9.38 and 15.92%, respectively (Figure 5D). However, IS significantly increased Pielou_e by 10.26%.

The distribution of microorganisms’ gene copies was shown in Table 2. At 0–20 cm, compared to RT, IS and IO significantly increased bacterial and fungal gene copies, while other treatments showed no significant effect. Specifically, bacterial gene copies increased by 26.09% under IS and 78.65% under IO; fungal gene copies increased by 18.43 and 101.44%, respectively. At 20-40 cm, microorganisms’ gene copies were lower than in the 0–20 cm soil. Compared with RT, the rice-eel system significantly increased bacterial and fungal gene copies by 51.60 and 71.42% on average. Moreover, IO reached the peak of bacteria and fungi gene copies, which were 93.38 and 96.70% higher than RT, respectively.

**Table 2:** Effects of different treatments on the biological properties of soil.

Treatments	0–20 cm		20–40 cm	
	Copies of bacteria (10^7^ copies g^−1^)	Copies of fungi (10^6^ copies g^−1^)	Copies of bacteria (10^7^ copies g^−1^)	Copies of fungi (10^6^ copies g^−1^)
RT	1.654E ± 1.28c	2.60E ± 0.28c	1.29E ± 0.95d	1.71E ± 0.47d
IRT	1.903E ± 0.80bc	2.76E ± 0.40bc	1.54E ± 0.41c	2.75E ± 0.71b
I70	1.871E ± 0.16bc	2.40E ± 0.78c	1.73E ± 0.50c	2.36E ± 0.47c
IS	2.09E ± 1.67b	3.08E ± 0.40b	2.06E ± 1.23b	3.22E ± 0.08a
IO	2.96E ± 0.10a	5.24E ± 0.07a	2.50E ± 0.95a	3.36E ± 0.30a

Different lowercase letters in the same column of data indicate significant differences among the data *p* < 0.05.

### 3.4 Soil microbial community composition

**Figure 6:**
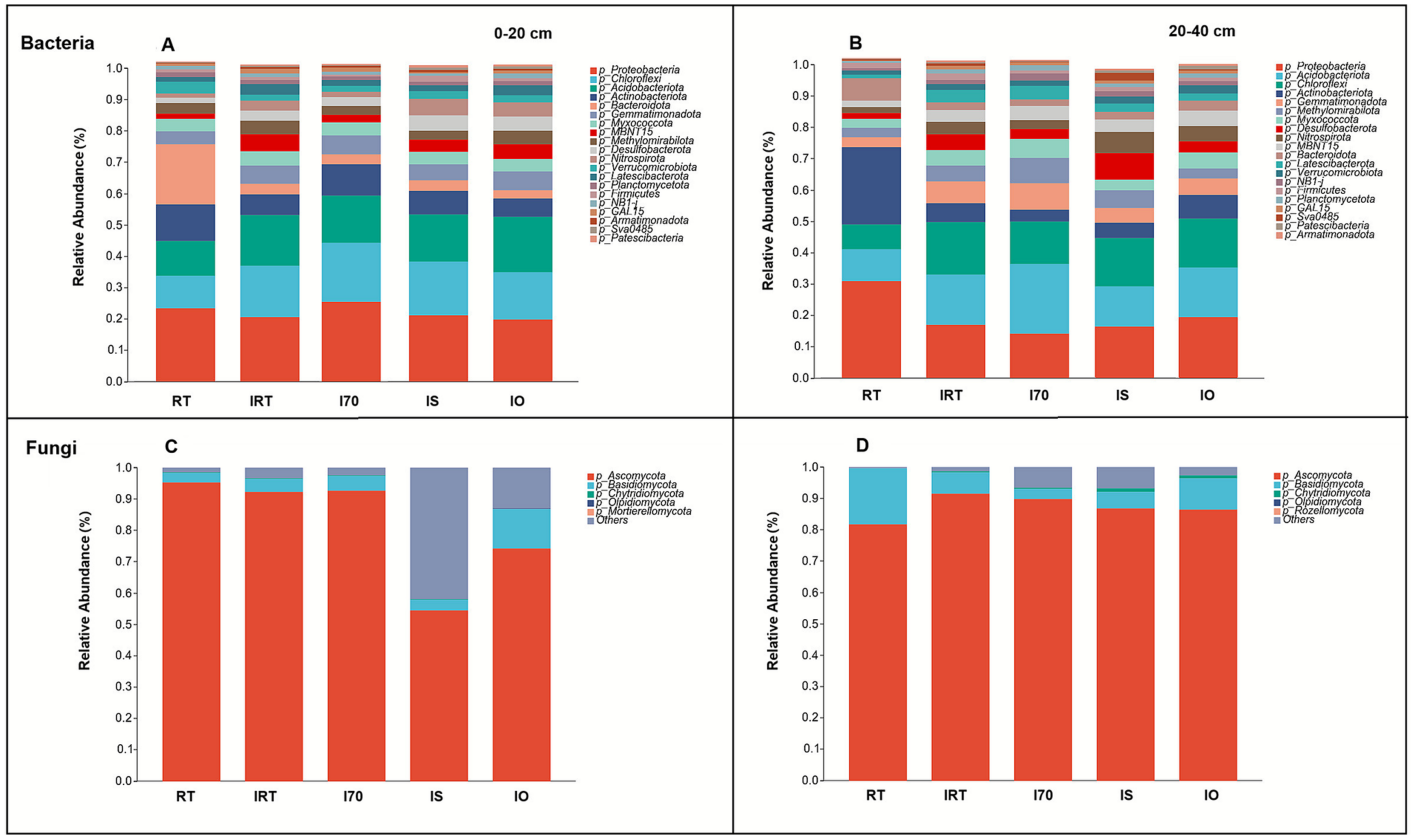
The effects of rice-eel system on bacterial and fungal community composition at different depths. The bacterial and fungal community composition is shown in **(A–D)**, respectively. Panels **(A,C)** indicate 0–20 cm soil, **(B,D)** indicate 20–40 cm soil.

Figures 6A,B show the top 10 bacterial phylum in two soil layers. Proteobacteria (21.23% at 0–20 cm and 18.82% at 20–40 cm), Chloroflexi (14.89% at 0–20 cm and 13.34% at 20–40 cm), and Acidobacteriota (14.40% at 0–20 cm and 14.72% at 20–40 cm) dominated the communities. At 0–20 cm, compared with RT, the rice-eel system significantly increased the relative abundance of Chloroflexi (45.69–82.80%), Acidobacteriota (35.03–58.13%), Gemmatimonadota (25.24–51.87%), MBNT15 (39.03–152.82%), and Desulfobacterota (52.62–178.54%) (Figure 6A) (Supplementary Table S1). Conversely, the relative abundance of Actinobacteriota and Bacteroidota significantly decreased by 15.12–49.11% and 82.08–87.18%, respectively. For Proteobacteria, I70 significantly increased its relative abundance, while the other rice-eel system treatments significantly reduced it, with an average of 12.53% lower than RT. Compared with RT, IRT and IO significantly increased the relative abundance of Methylomirabilota by an average of 33.68%, while I70 and IS significantly decreased by an average of 15.48%. In the 20–40 cm soil, compared with RT, the rice-eel system significantly increased the relative abundances of Acidobacteriota (27.47–119.11%), Chloroflexi (70.79–109.62%), Gemmatimonadota (53.63–174.77%), Methylomirabilota (3.61–159.54%), Myxococcota (21.10–109.19%), Desulfobacterota (75.02–340.74%), Nitrospirota (50.66–278.24%), and MBNT15 (96.57–145.03%) (Figure 6B). Conversely, the rice-eel system significantly reduced the relative abundances of Proteobacteria and Actinobacteriota by 45.87 and 77.63% on average, respectively (Supplementary Table S3).

In both soil layers, the top 10 fungal phylum were shown in Figures 6C,D. Ascomycota and Basidiomycota dominated the fungal communities. At 0–20 cm, their average proportions in the soil fungal sequence data were 81.80 and 5.59%, respectively (Figure 6C and Supplementary Table S4). At 20–40 cm, these values increased to 87.28 and 8.58%, respectively (Figure 6D). In the 0–20 cm soil, compared with RT, IO significantly reduced the relative abundance of Basidiomycota by 299.16%, while IS significantly reduced Ascomycota by 42.72% and increased Chytridiomycota by 234.78% (Supplementary Table S4). There was no significant difference between Olpidiomycota and Mortierellomycota in different treatments. At 20–40 cm, compared with RT, the relative abundance of Basidiomycota was significantly increased by 72.16% on average under IRT, I70 and IS. IS and IO also significantly increased the relative abundance of Chytridiomycota by 1.14% on average. Other fungal phyla showed no significant differences among all the treatments.

### 3.5 Specific communities with significant differences

**Figure 7:**
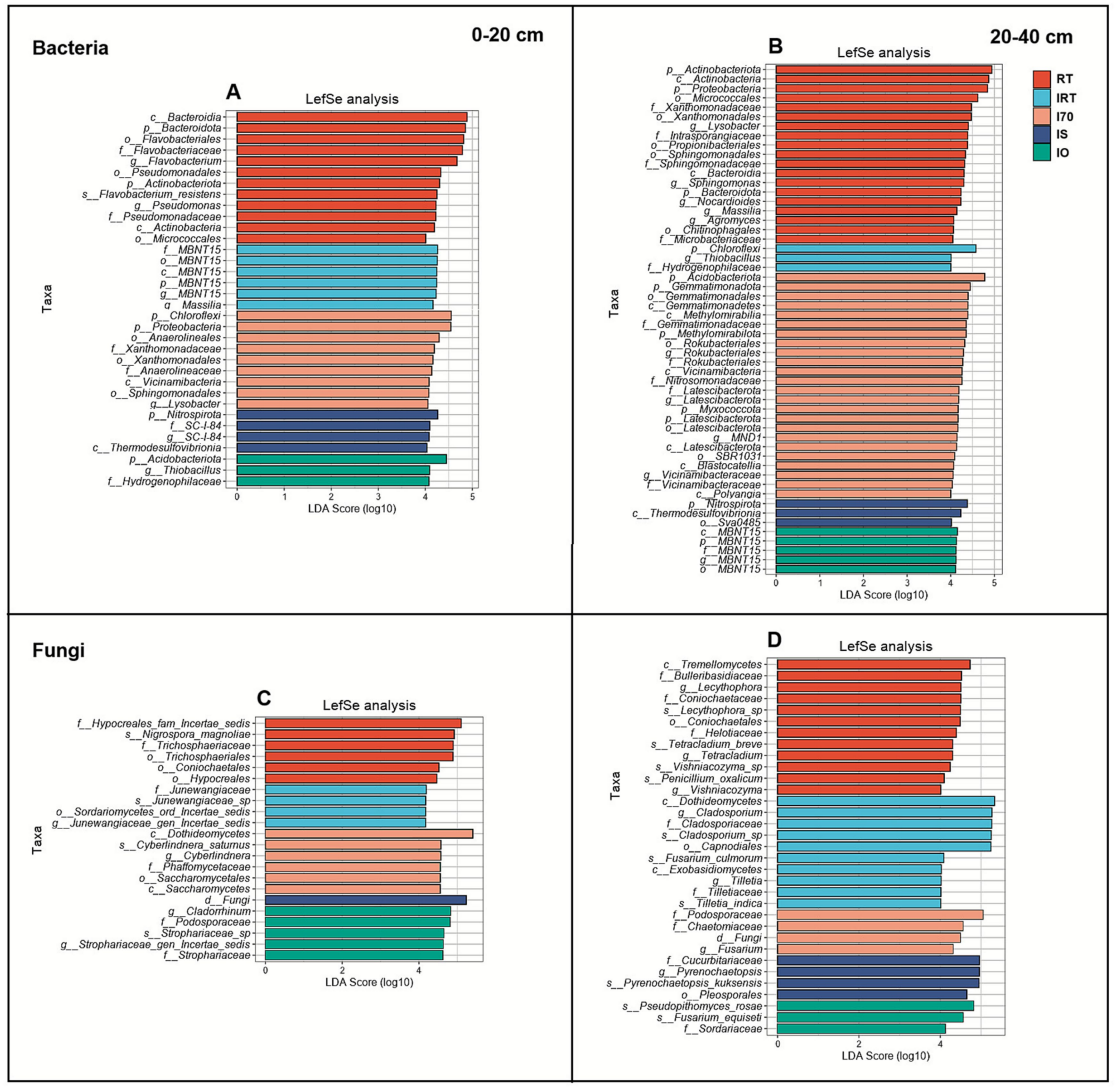
The effects of rice-eel system on bacterial and fungal LEfSe (the linear discriminant analysis effect size) at different depths. The bacterial and fungal LEfSe is shown in **(A–D)**, respectively. Panels **(A,C)** indicates 0–20 cm soil, **(B,D)** indicates 20–40 cm soil.

Linear Discriminant Analysis Effect Size (LEfSe) was used to identify significant differences in bacterial and fungal communities (Figures 7A–D). In 0–20 cm soil, total of 34 bacterial taxa had LDA scores greater than 4 (Figure 7A). Among these, 12 bacterial groups were enriched in RT. The IRT included only two types of bacteria: MBNT15 and Massilia (genus level). I70 showed enrichment of 9 bacterial taxa, representing the largest number of significantly different taxa among the rice-eel treatments. IS and IO had 4 and 3 bacterial taxa, respectively. Specifically, Nitrospirota (phylum level), SC_I_84 (family and genus level), and Thermodesulfovibrionia (class level) were enriched under IS, while Acidobacteriota (phylum level), Thiobacillus (genus level), and Hydrogenophilaceae (family level) were aggregated under IO. At 20–40 cm, 54 bacterial taxa had LDA scores greater than 4 (Figure 7B). RT remained the treatment with the highest number of significant difference taxa, with 19 taxa identified. Similar to the result of 0–20 cm soil, I70 had the most significantly different taxa among the rice–eel system treatments, with a total of 24 bacterial taxa. Both IRT and IS contained 3 taxa each. Under IRT, Chloroflexi (phylum level), Thiobacillus (genus level), and Hydrogenophilaceae (family level) were enriched. And under IS, Nitrospirota (phylum level), Thermodesulfovibrionia (class level), and Sva0485 (order level) were enriched. IO had only one type of bacteria, the MBNT15.

At 0–20 cm, 21 fungal taxa had LDA scores greater than 4 (Figure 7C). The distribution of fungal communities with significant phylogenetic differences were 2 at the class level,5 at the order level,6 at the family level, 4 at the genus level, and 4 at the species level. Among all treatments, I70 had the highest number of significantly different fungal taxa in the rice-eel system, with 6 taxa enriched: Dothideomycetes (class level), Cyberlindnera (genus and species level), Phaffomycetaceae (family level), and Saccharomycetales (order and class level). IRT and IO aggregated 4 and 5 fungal taxa, respectively. Under IRT Junewangiaceae (species, genus and family level) and Sordariomycetes (order level). And under IO, Podosporaceae (family level) and Strophariaceae (species, genus and family level) were dominant. At 20–40 cm, 32 fungal taxa had LDA scores greater than 4 (Figure 7D). IRT had the highest number of significantly different fungal taxa in the rice-eel system, with 10 taxa enriched. I70, IS and IO had 4, 4 and 3 taxa, respectively. Cucurbitariaceae (family level), Pyrenochaetopsis (species and genus level), and Pleosporales (order level) were enriched in IS. Under I70, Dothideomycetes (class level), Cladosporium (genus level), and Cladosporiaceae (species) showed the most significant differences. Podosporaceae (family level) and Pseudopithomyces (species level) showed the most significant differences under I70 and IO, respectively.

### 3.6 The key environmental factors related to microbial communities

**Figure 8:**
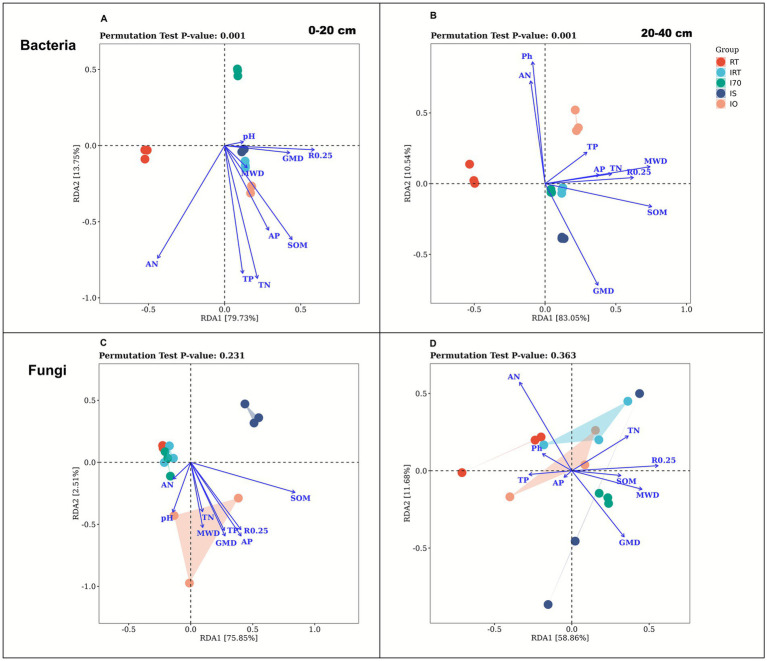
The effects of rice-eel system on redundancy analysis (RDA). The bacterial community structure is shown in **(A,B)**. The fungal community structure is shown in **(C,D)**. Panels **(A,C)** indicates 0–20 cm soil, **(B,D)** indicates 20–40 cm soil.

Redundancy Analysis (RDA) was conducted to assess the relationships between soil physicochemical properties and microbial community structure (Figure 8). At both 0–20 cm and 20–40 cm soil, soil properties explained93.48 and 93.59% of the bacterial community variation, respectively. RDA1 accounted for 79.73% (0–20 cm) and 83.05% (20–40 cm), while RDA2 explained 13.75 and 10.54%, respectively. Compared with RT, the rice-eel system significantly altered bacterial communities in both layers (*p* = 0.001), the bacterial community structures of the rice-eel system and RT were significantly different, and there were also differences in the community structure among various rice-eel system treatments. At 0–20 cm, R0.25, GMD and SOM were the primary factors of bacterial community. At 20–40 cm, SOM, MWD and R0.25 were dominant. In the two soil layers, soil properties explained 78.36% (0–20 cm) and 70.54% (20–40 cm) of the fungal community variation, respectively. Respectively. RDA1 accounted for 78.85% (0-20 cm) and 58.86% (20–40 cm) and RDA2 accounted for 2.51% (0-20 cm) and 11.86% (20–40 cm). At 0–20 cm, the fungal community structures of IS and IO differed from those of other treatments, among which the community structure of IO had the highest degree of dispersion. SOM, R0.25 and AP were the main environmental influencing factors of the fungal community at this soil layers. At 20–40 cm, except for IRT, the fungal community structure of other rice-eel system treatments differed from that of RT, and R0.25, MWD and GWD were the main factors. Overall, all the treatments had no significant effect on the fungal community (*p* > 0.05), but had a significant effect on the bacterial community, indicating that the rice-eel system had a stronger effect on soil bacteria than fungi. SOM and R0.25, as the main factors, affected the structure of bacterial and fungal communities.

Given the variation in soil structure and chemical property improvements under different fertilization treatments in the rice-eel system, grey relational analysis (GRA) was used to comprehensively assess the effectiveness of each treatment. Eight indicators—SOM, TN, TP, AN, AP, R0.25, GWD, MWD, bacterial and fungal Simpson, Pielou_e, gene copies—were selected, along with five treatments, to construct the grey system for evaluating each treatment on soil structure and soil nutrient content in both the 0–20 cm and 20–40 cm soil layers (Table 3). A higher Grey Relational Degree (GRD) indicates a stronger soil improvement effect. Table 3 is better. The overall ranking of treatments was: IO > IS > IRT > I70 > RT. In the rice-eel system, all treatments showed better soil improvement than RT. Treatments combining reduced nitrogen with straw and organic fertilizer (IS and IO) demonstrated greater effectiveness than conventional fertilization (IRT) and 70% fertilization treatment (I70). Notably, IO showed the most significant improvement in both soil structure and nutrient content.

**Table 3:** Grey Relation Degree of soil chemical properties and water stability of soil aggregates under various treatments.

Treatment	GRD	Sort result
RT	0.861	5
IRT	1.090	3
I70	0.991	4
IS	1.267	2
IO	1.391	1

GRD, Grey Relation Degree.

### 3.7 Microbial community function prediction

**Figure 9:**
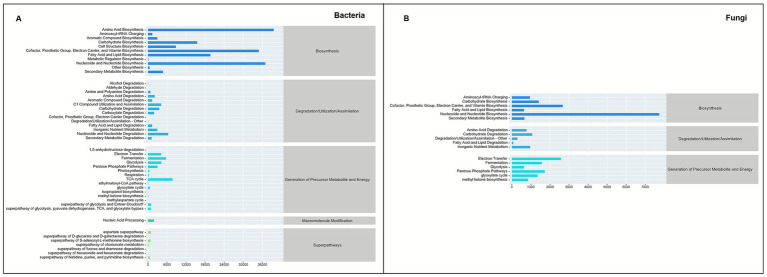
Functional prediction results of different bacterial and fungal communities of PICRUSt2 in rice-eel system soil based on MetaCyc database. Panels **(A,B)** indicates bacterial and fungal communities respectively.

PICRUSt2 was employed to predict the functional profiles of bacterial and fungal communities in the rice-eel system under different fertilization regimes. At hierarchy level 1, the bacterial community comprised 5 functional categories, ranked by relative abundance as Biosynthesis, Degradation/Utilization/Assimilation, Generation of Precursor Metabolites and Energy, Macromolecule Modification, and Superpathways. The fungal community was dominated by 3 categories: Biosynthesis, Degradation/Utilization/Assimilation, and Generation of Precursor Metabolites and Energy (Figure 9).

**Figure 10:**
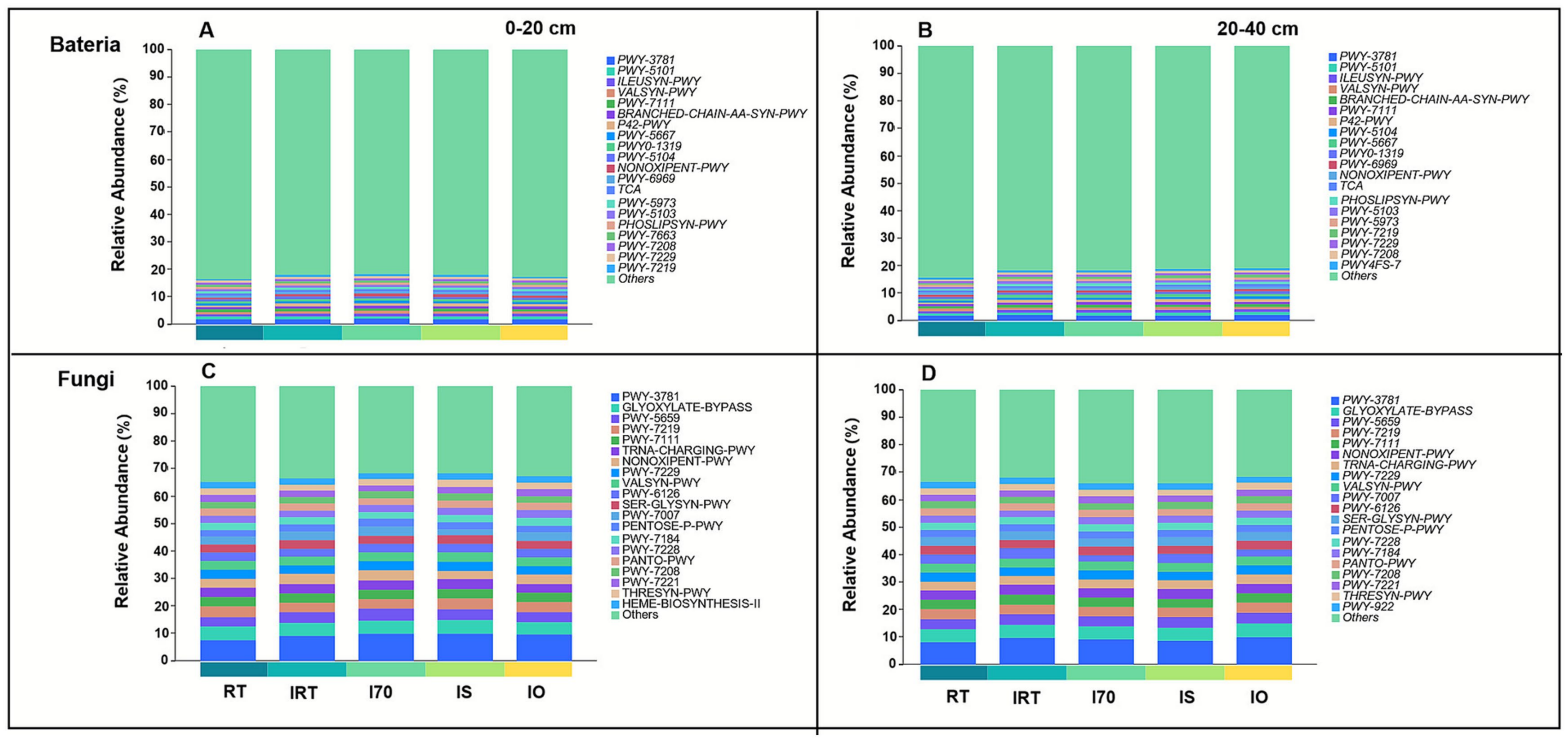
The effects of rice-eel system on microbial function prediction. The bacterial community function is shown in **(A,B)**. The fungal community function is shown in **(C,D)**. Panels **(A,C)** indicates 0–20 cm soil, **(B,D)** indicates 20–40 cm soil.

In both soil layers, 20 functional units were identified for each community (Figure 10). The predominant bacterial metabolic pathways included aerobic respiration I-cytochrome c (PWY-3781), pyruvate fermentation to isobutanol (PWY-7111), and L-isoleucine biosynthesis (ILEUSYN-PWY), with average relative abundances of 1.84, 0.97, and 0.92%, respectively (Figures 10A,B). At 0–20 cm, compared with RT, the rice-eel system significantly reduced the relative abundance of bacteria involved in fatty acid metabolism (PWY-5973) and gondoate biosynthesis (PWY-7663) (Figure 10A and Supplementary Table S5). In contrast, it enhanced the relative abundances of most other pathways, peaking under the IO. Specifically, the relative abundances of PWY-3781 (4.40%), PWY-5101 (2.72%), and ILEUSYN-PWY (2.09%) under IO were significantly higher than those under RT. At 20–40 cm, compared to RT, the rice-eel system significantly increased the relative abundance of bacteria across all 20 pathways (Figure 10B). In particular, the relative abundances of PWY-5101 and ILEUSYN-PWY were the highest under IS, exceeding those in RT by 17.62 and 16.25%, respectively (Supplementary Table S5).

For fungi, the major metabolic pathways were aerobic respiration I – cytochrome c (PWY-3781), glyoxylate bypass and GDP-sugar biosynthesis (PWY-5659) (Figures 10C,D). In the two layers of soil, the average relative abundances in the fungal community were 9.07, 4.78 and 3.82%, respectively. At 0–20 cm, compared to RT, the rice-eel system significantly increased the relative abundance of fungi involved in both PWY-3781 and PWY-5659, and the highest abundance observed under IO—24.88 and 7.45% greater than RT, respectively (Figure 10C and Supplementary Table S6). In contrast, at 20–40 cm, the influence of the rice-eel system on fungal metabolic pathways was minimal (Figure 10D).

## 4 Discussion

### 4.1 Effect of rice-eel system on soil nutrient cycling

**Figure 11:**
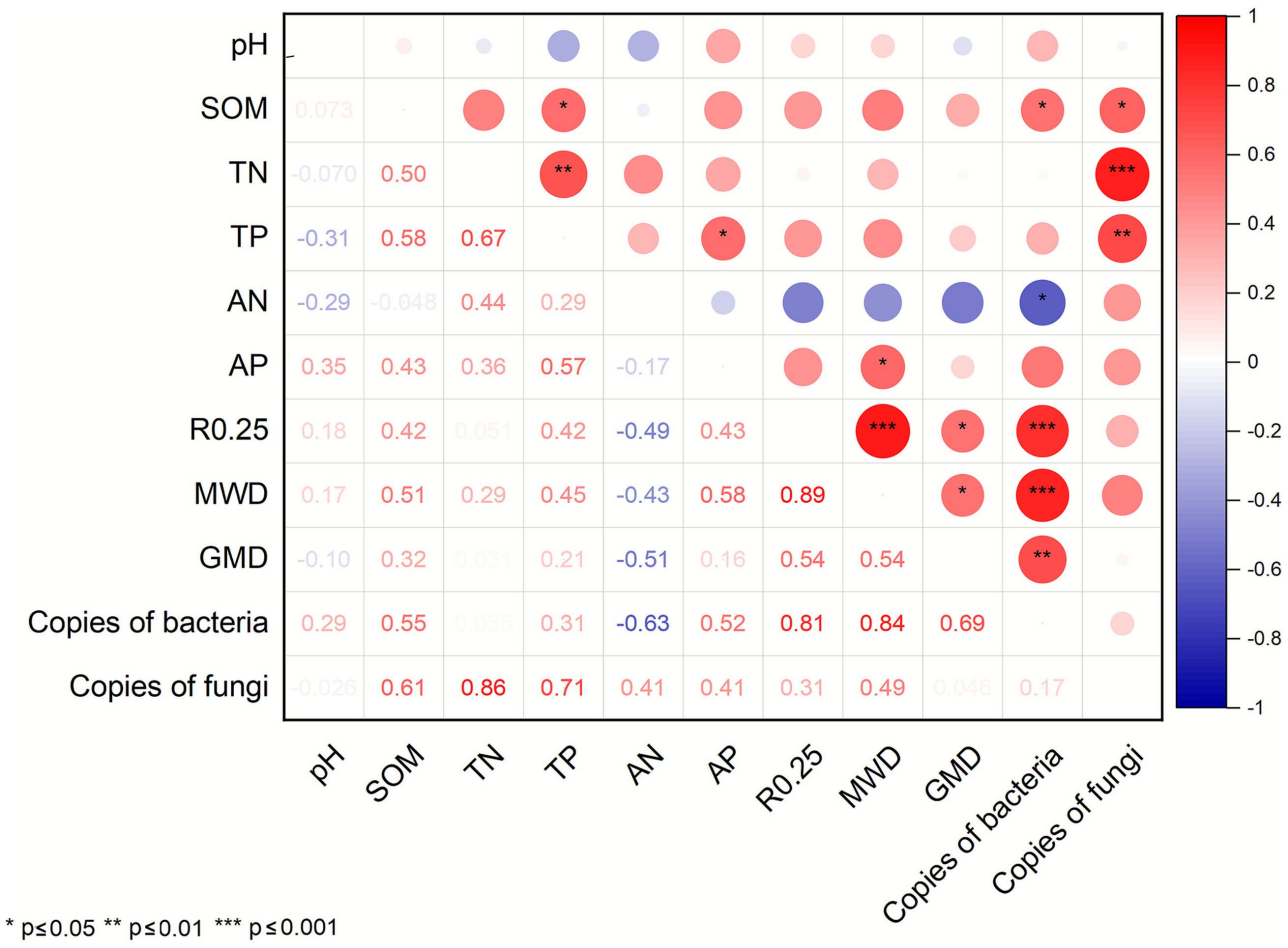
The correlation analysis between soil physical and chemical properties and soil microbial gene copies.

Soil nutrient contents play a key role in plant growth, agricultural productivity, and environmental sustainability. At 0–20 cm, compared with RT, IO significantly increased the contents of SOM (15.09%), TN (7.27%), TP (15.05%), and AP (34.92%), while IS showed the highest SOM content, 16.98% greater than RT. In contrast, I70 had the lowest contents of SOM, TN, TP, and AN, reflecting the limitation of reduced fertilization on nutrient accumulation. These results demonstrate that substituting chemical fertilizer with straw and organic fertilizer under the rice–eel system is an effective strategy for enhancing soil nutrient supply. Straw and organic fertilizer inherently improve soil nutrient content due to their composition and characteristics (Xue et al., 2024; Yan et al., 2025). Moreover, eel excrement is rich in nitrogen and phosphorus and contains abundant microorganisms. Together with eel burrowing activity, it improves soil permeability and creates favorable conditions for microbial proliferation (Zandonà et al., 2021). These biotic processes jointly accelerate the decomposition and transformation of organic inputs, thereby enhancing nutrient availability and sustaining soil productivity, consistent with previous findings (Mariyono, 2024; Wiesebron et al., 2021; Zandonà et al., 2021). Functional predictions based on PICRUSt2 further corroborated the enhanced microbial metabolic potential associated with nutrient cycling (Figure 9). Soil nutrient dynamics, regulated by microbial processes such as mineralization and organic matter decomposition, are also influenced by fertilization rate (Ghatak and Ansar, 2017; Mazur Z and Mazur T, 2015). Figure 11 also shows that the copy number of microbial genes is significantly positively correlated with nutrient indicators such as SOM, and a series of metabolic pathways of microorganisms are also related to soil nutrient cycling. Consequently, despite belonging to the rice-eel system, I70 provided lower nutrient availability than RT because of reduced fertilization input. Furthermore, the higher SOM and AP contents observed under IS and IO, together with changes in deeper soil layers, suggest that eel activity exerts both direct and indirect effects on vertical nutrient redistribution. These effects are likely driven by bioaccumulation, aggregate-associated nutrient protection, and shifts in microbial community structure (Marschner and Rengel, 2023; Zhao et al., 2022). Overall, substituting chemical fertilizer with straw and organic fertilizers under the rice-eel system improved nutrient availability and reduced reliance on chemical fertilizer.

### 4.2 Effect of rice-eel system on soil structure

Soil aggregates are key indicators of soil quality, as their size distribution and stability directly influence gas exchange, water infiltration, erosion resistance, and carbon sequestration (Han, et al., 2021; Pereira et al., 2021). In this study, the rice-eel system promoted macroaggregate formation (>0.25 mm) while reducing microaggregates (<0.053 mm). Specifically, IO significantly increased R0.25 by 39.88% compared to RT, indicating a substantial improvement in soil structure, consistent with higher SOM and aggregate stability under IO. Since macroaggregates (>0.25 mm) are associated with better soil structure and nutrient retention (Wang et al., 2022), this highlights the structural advantages of the rice-eel system. GRD analyses further confirmed that IO had the strongest effect, indicating that eel bioturbation combined with organic matter inputs jointly enhanced nutrient availability and aggregate stabilization, resulting in the most pronounced soil structural improvement. Eel feces contribute calcium precipitation and nutrient cycling functions that facilitate macroaggregate formation (Fan et al., 2023; Mekuchi et al., 2010; Tian et al., 2022). Eel burrowing stimulates microbial biomass, and microbes secrete extracellular polysaccharides that cement soil particles into stable aggregates (Sun et al., 2024). In addition, increased SOM derived from straw and organic fertilizer interacts with fine particles and iron oxides, further stabilizing aggregates (Ren et al., 2024; Zhao et al., 2019). In the 20–40 cm soil layer, IS and IO significantly increased R0.25 and MWD, confirming that the positive effects of eel activity and organic inputs extend beyond the surface layer. Aggregate stability, as reflected in MWD and GMD, was positively correlated with SOM and nutrient accumulation, indicating that vertical redistribution of organic matter, primarily facilitated by eel bioturbation, plays a critical role in deep-soil aggregation, with microbial activity potentially contributing to aggregate stabilization (Khosravani et al., 2024; Zhang et al., 2021).

Overall, the rice-eel system promoted the transformation of microaggregates into stable macroaggregates, enhanced aggregate stability, and improved soil structure, with IO showing the strongest effect due to the combined influence of readily available organic matter and the biological activity associated with straw.

### 4.3 Effect of rice-eel system on the diversity of the microbial community

Microbial communities are fundamental to soil ecosystem functioning because they regulate nutrient turnover, organic matter decomposition, and aggregate stability (Si et al., 2017; Wu et al., 2024). In this study, at 0–20 cm, compared with RT, IS and IO significantly increased bacterial and fungal gene copies. At 20–40 cm, all treatments under the rice-eel system significantly increased bacterial and fungal gene copies. In both soil layers, compared with RT, the rice-eel system significantly increased bacterial Simpson and Pielou_e indices. For fungal communities, IO (0–20 cm) and IS (20–40 cm) significantly increased fungal Pielou_e. This improvement in community diversity and gene copy abundance highlights the positive impact of eel activity combined with organic inputs on bacterial and fungal communities. These effects can be attributed to multiple mechanisms. Increased SOM, TN, and other nutrients provide diverse substrates that support microbial proliferation (Wu et al., 2024). Figure 11 also confirms this point. The significant positive correlations between TN, TP and fungal gene copy numbers, as well as between SOM and both bacterial and fungal gene copy numbers, indicate that nutrient enrichment under the rice–eel system directly supports microbial proliferation, thereby strengthening microbially mediated metabolic pathways involved in organic matter decomposition and nutrient transformation. Nitrogen reduction strategies can reduce the competitiveness of nitrogen-sensitive taxa, thereby removing ecological niche restrictions on weaker competitive taxa and diversifying the community. Organic substitutes such as straw and organic fertilizer promote microbial diversity through decomposition and biochemical transformation processes, supplying labile carbon and nutrients that stimulate microbial growth (Lunstrum and Chen, 2014; Wu et al., 2024). In addition, eel feces directly contribute microbial biomass, while burrowing activity enhances oxygen diffusion and creates heterogeneous niches favorable to aerobic and facultative microbes (Fan et al., 2023; Li J. et al., 2021; Li X. et al., 2021; Tian et al., 2022; Yuan et al., 2023). Eels feed on earthworms (Itakura et al., 2021), and their reduced presence may lead to organic matter accumulation, further enriching the soil environment for bacteria and fungi (Ferlian et al., 2020; Lv et al., 2016). Earthworms also produce antimicrobial peptides that suppress certain microbial populations. A reduction in earthworm activity lowers peptide levels, potentially enabling the establishment of more diverse microbial communities (Chang et al., 2016).

Overall, the rice-eel system promoted microbial diversity through a combination of nutrient enrichment, bioturbation, and trophic interactions. Substituting chemical fertilizer with straw and organic amendments provided sustained nutrient inputs and diverse carbon sources, which stimulated microbial growth and functional diversification. This organic substitution enhanced soil fertility and fostered more resilient microbial communities, thereby strengthening microbially mediated soil functions.

### 4.4 Effect of rice-eel system on the composition of the bacterial community

In the 0–20 cm soil layer, the rice–eel system significantly increased the relative abundances of Chloroflexi, Acidobacteriota, Gemmatimonadota, and Desulfobacterota. Chloroflexi and Gemmatimonadota are dominant in paddy soils and flooded agroecosystems, actively participating in organic matter decomposition and nutrient turnover under low-oxygen conditions (Xue et al., 2020; Yarwood, 2021). Gemmatimonadota, which is often enriched within soil aggregates, showed its highest relative abundance under IO, consistent with the observed enhancement of macroaggregate formation in this treatment. Acidobacteriota is widely distributed in paddy soils and is involved in nitrogen fixation, extracellular polysaccharide (EPS) synthesis, phosphorus solubilization, and degradation of plant-derived polymers, all essential for nutrient cycling (Duan et al., 2022; Gonçalves et al., 2024). EPS acts as a cementing agent that contributes to aggregate stability (Sun et al., 2024). Accordingly, the highest abundance of Acidobacteriota occurred under IO, in line with the observed increases in SOM and macroaggregates. Notably, Acidobacteriota, which represented a substantial proportion of the bacterial community in this study, have been increasingly recognized as key responders to soil carbon availability and redox conditions in flooded rice systems. Amplicon-based and metagenomic studies in long-term paddy soils demonstrate that Acidobacteriota are closely associated with soil organic carbon dynamics and nutrient status, suggesting their involvement in anaerobic carbon turnover and adaptive responses to fluctuating moisture and nutrient inputs (İnceoğlu et al., 2013; Luo et al., 2023; Reardon et al., 2022). The enrichment of Desulfobacterota reflects enhanced sulfate-reducing activity under anaerobic conditions. In flooded agricultural soils, members of Desulfobacterota function as sulfate-reducing bacteria that drive sulfur cycling and facilitate the anaerobic mineralization of organic matter, thereby coupling carbon and sulfur biogeochemical processes. Recent reviews and paddy soil studies confirm that these taxa play a central role in maintaining redox balance and regulating organic matter decomposition in waterlogged environments (Diao et al., 2023). By contrast, I70 significantly increased the relative abundance of Proteobacteria, likely reflecting their strong competitive advantage under nutrient-limited conditions. Proteobacteria are known for their rapid utilization of available carbon and nutrients and their association with enzymatic activities related to nitrogen and phosphorus cycling in agricultural soils (Yang et al., 2023; Liu et al., 2021; Xu et al., 2020). However, the addition of exogenous organic matter in IS and IO treatments alleviated nutrient limitation and reduced this competitive dominance, allowing other functional taxa to proliferate (Zhang et al., 2019). The LEfSe analysis results show that Nitrospirota accumulates under IS, likely due to straw incorporation, which increases the availability of organic carbon and nutrients essential for its growth (Chen et al., 2025; Luo et al., 2017). Under IO, Thiobacillus was enriched and is known to produce EPS and lipopolysaccharides that act as soil-binding agents, thereby enhancing particle adhesion, aggregate stability, and reducing erosion risk (Cania et al., 2020a,b). These microbial responses are likely facilitated by eel bioturbation and the downward transport of organic matter, creating more favorable conditions for microbial activity, nutrient availability, and aggregate formation throughout the soil profile.

At 20–40 cm, compared with RT, the rice-eel system significantly increased the average relative abundances of Acidobacteriota (65.64%), Chloroflexi (92.58%), Gemmatimonadota (106.04%), and Desulfobacterota (168.39%). This indicates that the rice-eel system enhances organic matter decomposition and promotes nutrient cycling in subsurface soil layers (Duan et al., 2022; Gonçalves et al., 2024; Reardon et al., 2022). The rice-eel system also significantly increased the relative abundance of Nitrospirota, Myxococcota and Methylomirabilota. Nitrospirota, is involved in soil nitrogen transformations, converting nitrogen into forms available to plants or other microbes (Han et al., 2021; Li J. et al., 2021; Li X. et al., 2021). Myxococcota contributes to the nitrogen cycle through nitrogen fixation and the degradation of nitrogenous compounds (Chen et al., 2023; Jia et al., 2025). Methylomirabilota supports phosphorus acquisition, iron chelation, plant hormone production, and plant growth promotion (Kumar et al., 2016). The LEfSe analysis results showed that the enrichment of Nitrospirota observed in IS at 0–20 cm also extends to the 20–40 cm layer, indicating that the addition of organic fertilizers and the bioturbation effect of eels facilitate the vertical continuity and activity of microbial communities in subsurface soil.

In summary, the rice-eel system restructured bacterial communities by selectively enriching taxa involved in nutrient cycling, organic matter decomposition, nitrogen fixation, and soil aggregation. Eel activity and organic inputs jointly create favorable physical and chemical conditions, which, together with microbial metabolism, enhance nutrient availability and aggregate stabilization throughout the soil profile. These changes enhanced the overall function of the bacterial community, particularly through pathways such as aerobic respiration (cytochrome c pathway) and pyruvate fermentation to isobutanol. As a result, bacteria gained greater ability to decompose organic matter and promote nutrient cycling, thereby increasing nutrient availability. Bacteria also produced secondary metabolites and cementing agents that facilitated soil particle aggregation and improved soil structural stability. These findings were consistent with the RDA results, which identified R0.25, GMD, MWD, and SOM as the primary environmental factors influencing bacterial communities in both soil layers (Figure 8). Notably, IO and IS treatments, through the addition of exogenous organic matter, significantly improved soil nutrient content and provided substrates for bacterial metabolism, while also promoting soil structural stability and further shaping bacterial community composition.

### 4.5 Effect of rice-eel system on the composition of the fungal community

At 0–20 cm, IO significantly reduced the relative abundance of Basidiomycota and IS significantly reduced that abundance of Ascomycota. Basidiomycota produce hydrolytic enzymes, such as β-glucosidase and cellobiohydrolase, which promote the decomposition of organic matter and nutrient mineralization (Crowther et al., 2011; Šnajdr et al., 2011). Ascomycota also plays a key role in soil ecosystems by facilitating carbon and nitrogen cycling through organic matter decomposition (Challacombe et al., 2019). SOM and TN levels in IO and IS were significantly higher than those in RT. This may be due to the decreased fungal abundance slowing organic matter decomposition, leading to its accumulation and gradual release of nutrients over time (Challacombe et al., 2019; Morrison et al., 2016). LEfSe analysis revealed that Dothideomycetes and Saccharomycetales were enriched in I70. Dothideomycetes influence microbial interactions and soil particle aggregation (Frouz, 2013). Saccharomycetales aid in organic matter mineralization and facilitate the formation and stabilization of soil microaggregates (Botha, 2011). These results were mutually corroborated with the increased content of microaggregates under I70. Cladorrhinum and Strophariaceae were enriched in IO. Cladorrhinum, an arbuscular mycorrhizal fungus (AMF), enhances aggregate stability and slows the turnover of macroaggregates. Strophariaceae decomposes lignocellulosic substrates, contributing to carbon sequestration, and secrete extracellular polymers that bind soil particles, improving soil structure (Dou et al., 2025; Gadd et al., 2007). The mycelial network of Strophariaceae also attracts soil invertebrates, further promoting nutrient cycling (Chen et al., 2024). This result corresponds with increased SOM, the number of macroaggregates, MWD, and GWD.

At 20–40 cm, IS and IO significantly increased the relative abundance of Chytridiomycota. Chytridiomycota promotes the decomposition of organic matter, particularly polymeric substances, and facilitates the solubilization and utilization of various phosphorus sources, including orthophosphate, phytic acid, and hydroxyapatite (Hanrahan-Tan et al., 2022). The LEfSe analysis results showed that Pleosporales enriched in IS. Pleosporales participate in cellulose degradation and facilitate straw decomposition and utilization (Ohm et al., 2012). Sordariaceae enriched in IO. Sordariaceae contributes to the organic nutrient cycle, and their abundance was positively correlated with SOM content, which corroborates the observation that SOM under IS was significantly higher than in pure chemical fertilizer treatments (Zhang et al., 2019). All of results suggest that different fertilization regimes under the rice-eel system selectively shape fungal community composition and function.

In summary, the rice-eel system significantly altered the fungal community composition by increasing the abundance of fungi involved in soil aggregate formation, cellulose decomposition, and nutrient cycling, enhancing functional pathways such as aerobic respiration (cytochrome c pathway) and the glyoxylate bypass. Under this system, fungi interacted with other soil microorganisms to form a complex ecological network that strengthens nutrient cycling and mineralization (Koshila Ravi et al., 2019; Yan et al., 2025). The rice-eel system also stimulates the production of extracellular polymers and the expansion of mycelial networks, which bind soil particles and support the formation of stable aggregates (Baruah and Sahu, 2019; Singh et al., 2022). Soil particles were further bound by cementing substances such as organic matter, enhancing aggregate stability and reinforcing the structural integrity and ecological function of the soil. The RDA results corroborated these findings, identifying SOM, R0.25, MWD, and GWD as key factors influencing fungal community composition across both soil layers (Figure 7). Notably, the addition of exogenous organic matter has intensified the influence of IO and IS on the fungal community.

### 4.6 Effect of rice-eel system on the microbial community function prediction

Bacterial and fungal communities in the rice-eel system exhibited distinct yet complementary functional responses under different fertilization regimes. Bacteria showed broad enhancements in metabolic potential, with significant increases across most pathways, particularly at 20–40 cm, where all 20 functions were elevated. In contrast, fungi displayed a more selective response, with pronounced stimulation of aerobic respiration and glyoxylate bypass at 0–20 cm, but minimal changes at 20–40 cm. These patterns suggest that bacteria drive the expansion of metabolic diversity and nutrient turnover throughout the soil, while fungi primarily reinforce organic matter decomposition and aggregate stabilization near the surface. Together, these complementary shifts highlight the synergistic role of bacteria and fungi in maintaining soil fertility and structural stability under the rice-eel system (Hu et al., 2011; Li et al., 2025; Soong et al., 2018).

## 5 Conclusion

This study demonstrates that integrating rice-eel system enhances soil properties and microbial functionality compared with conventional treatment (RT). First, the rice-eel system improved soil nutrient availability, structure stability, and microbial diversity relative to RT, highlighting the synergistic effects of bioturbation and organic inputs. Second, within the rice-eel system, substituting chemical fertilizers with straw and organic fertilizer as part of this cultivation practice further promoted macroaggregate formation, nutrient retention, vertical redistribution of nutrients, and microbial functional potential, including roles in nutrient cycling, organic matter decomposition, phosphorus solubilization, cellulose degradation, and the synthesis of extracellular polymers that stabilize soil structure. Third, among the organic substitution treatments, differences organic matter composition led to variation in soil and microbial responses: compared with IS, IO exhibited superior nutrient supply, structural improvement, and microbial functional enrichment, indicating that the type and quality of organic inputs influence the magnitude of soil-microbe interactions. Overall, these findings provide novel evidence that the rice-eel system coupled with organic fertilizer substitution establishes a positive feedback loop among nutrients, soil structure, and microbial processes, enhancing soil multifunctionality, reducing chemical fertilizer dependency, and offering a farming method for sustainable rice production and long-term agroecosystem stability.

## Data Availability

The datasets presented in this study can be found in online repositories. The names of the repository/repositories and accession number(s) can be found at: https://www.ncbi.nlm.nih.gov/, PRJNA1321484.
