# Supplementary material for: Rice-eel system combined with exogenous organic waste improves soil quality under nitrogen deficiency by regulating soil microbial community
Source: Front Microbiol. 2026 Jan 14;16:1743071. doi: 10.3389/fmicb.2025.1743071 (PMC12847270; doi:10.3389/fmicb.2025.1743071)
Supplement: Supplementary file 1 [file Table_1.DOCX]

**Supplementary table S1** The dimensional value used for grey relational degree analysis

| Soil depth  (cm) | Treatments | SOM | TN | AN | TK | TP | AP | AK | R0.25 | MWD | GWD | bacterial Simpson | bacterial Pielou_e | Bacterial copie**s** | fungal Simpson | fungal Pielou_e | fungal copie**s** |
| --- | --- | --- | --- | --- | --- | --- | --- | --- | --- | --- | --- | --- | --- | --- | --- | --- | --- |
| 0-20 | RT | 0.474 | 0.431 | 0.000 | 0.685 | 0.478 | 0.440 | 0.737 | 0.553 | 0.238 | 0.504 | 1.000 | 1.000 | 0.782 | 0.748 | 0.789 | 0.490 |
|  | IRT | 0.316 | 0.000 | 0.241 | 0.189 | 0.369 | 0.480 | 0.733 | 0.353 | 0.243 | 0.397 | 0.194 | 0.041 | 0.633 | 0.701 | 0.906 | 0.752 |
|  | I70 | 0.579 | 0.845 | 1.000 | 0.447 | 0.981 | 0.520 | 1.000 | 0.154 | 0.170 | 0.174 | 0.140 | 0.058 | 0.652 | 0.804 | 0.632 | 0.873 |
|  | IS | 0.000 | 0.414 | 0.556 | 0.000 | 0.460 | 0.300 | 0.071 | 0.155 | 0.208 | 0.220 | 0.025 | 0.019 | 0.000 | 0.612 | 0.375 | 0.464 |
|  | IO | 0.053 | 0.086 | 0.222 | 0.129 | 0.000 | 0.000 | 0.000 | 0.000 | 0.000 | 0.000 | 0.147 | 0.045 | 0.522 | 0.000 | 0.000 | 0.103 |
| 20-40 | RT | 1.000 | 1.000 | 0.574 | 0.695 | 0.940 | 0.780 | 0.845 | 1.000 | 1.000 | 1.000 | 0.880 | 0.496 | 1.000 | 1.000 | 1.000 | 1 |
|  | IRT | 0.526 | 0.609 | 0.556 | 0.295 | 0.649 | 0.740 | 0.821 | 0.868 | 0.867 | 0.879 | 0.195 | 0.056 | 0.848 | 0.699 | 0.681 | 0.231 |
|  | I70 | 0.895 | 1.000 | 0.840 | 1.000 | 1.000 | 1.000 | 0.960 | 0.921 | 0.930 | 0.913 | 0.000 | 0.172 | 0.739 | 0.815 | 0.589 | 0 |
|  | IS | 0.474 | 0.776 | 0.926 | 0.060 | 0.513 | 0.460 | 0.789 | 0.770 | 0.791 | 0.747 | 0.088 | 0.026 | 0.536 | 0.571 | 0.545 | 0.262 |
|  | IO | 0.579 | 0.707 | 0.463 | 0.362 | 0.282 | 0.480 | 0.841 | 0.726 | 0.734 | 0.991 | 0.452 | 0.000 | 0.276 | 0.532 | 1.000 | 0.885 |

**Note:** SOM, soil organic matter; AP, available phosphorus; AN, available nitrogen; TP, total phosphorus; TN, total nitrogen; R0.25, the number of water-stable large aggregates, MWD, average weight diameter, GMD, geometric mean diameter
