# Supplementary material for: Rice-eel system combined with exogenous organic waste improves soil quality under nitrogen deficiency by regulating soil microbial community
Source: Front Microbiol. 2026 Jan 14;16:1743071. doi: 10.3389/fmicb.2025.1743071 (PMC12847270; doi:10.3389/fmicb.2025.1743071)
Supplement: Supplementary file 2 [file Table_2.DOCX]

**Supplementary table S2** The grey relation coefficient used for grey relational degree analysis

| Soil depth  (cm) | Treatments | SOM | TN | AN | TP | AP | R0.25 | MWD | GWD | bacterial Simpson | bacterial Pielou_e | fungal Simpson | fungal Pielou_e | Bacterial copie**s** | fungal copie**s** |
| --- | --- | --- | --- | --- | --- | --- | --- | --- | --- | --- | --- | --- | --- | --- | --- |
| 0-20 | RT | 0.514 | 0.537 | 1.000 | 0.511 | 0.532 | 0.475 | 0.677 | 0.498 | 0.333 | 0.333 | 0.390 | 0.401 | 0.388 | 0.505 |
|  | IRT | 0.613 | 1.000 | 0.675 | 0.576 | 0.510 | 0.586 | 0.673 | 0.558 | 0.721 | 0.923 | 0.441 | 0.416 | 0.356 | 0.399 |
|  | I70 | 0.463 | 0.372 | 0.333 | 0.338 | 0.490 | 0.765 | 0.746 | 0.741 | 0.781 | 0.895 | 0.434 | 0.383 | 0.442 | 0.364 |
|  | IS | 1.000 | 0.547 | 0.474 | 0.521 | 0.625 | 0.763 | 0.706 | 0.694 | 0.952 | 0.963 | 1.000 | 0.450 | 0.572 | 0.519 |
|  | IO | 0.905 | 0.853 | 0.692 | 1.000 | 1.000 | 1.000 | 1.000 | 1.000 | 0.773 | 0.918 | 0.489 | 1.000 | 1.000 | 0.829 |
| 20-40 | RT | 0.333 | 0.333 | 0.466 | 0.347 | 0.391 | 0.333 | 0.333 | 0.333 | 0.362 | 0.502 | 0.333 | 0.333 | 0.333 | 0.333 |
|  | IRT | 0.487 | 0.451 | 0.474 | 0.435 | 0.403 | 0.366 | 0.366 | 0.363 | 0.719 | 0.900 | 0.371 | 0.417 | 0.423 | 0.684 |
|  | I70 | 0.358 | 0.333 | 0.373 | 0.333 | 0.333 | 0.352 | 0.350 | 0.354 | 1.000 | 0.744 | 0.404 | 0.380 | 0.459 | 1.000 |
|  | IS | 0.514 | 0.392 | 0.351 | 0.493 | 0.521 | 0.394 | 0.387 | 0.401 | 0.851 | 0.951 | 0.483 | 0.467 | 0.479 | 0.657 |
|  | IO | 0.463 | 0.414 | 0.519 | 0.639 | 0.510 | 0.408 | 0.405 | 0.335 | 0.525 | 1.000 | 0.644 | 0.485 | 0.333 | 0.361 |

**Note:** SOM, soil organic matter; AP, available phosphorus; AN, available nitrogen; TP, total phosphorus; TN, total nitrogen; R0.25, the number of water-stable large aggregates, MWD, average weight diameter, GMD, geometric mean diameter
