# Supplementary material for: Rice-eel system combined with exogenous organic waste improves soil quality under nitrogen deficiency by regulating soil microbial community
Source: Front Microbiol. 2026 Jan 14;16:1743071. doi: 10.3389/fmicb.2025.1743071 (PMC12847270; doi:10.3389/fmicb.2025.1743071)
Supplement: Supplementary file 3 [file Table_3.DOCX]

**Supplementary table S3** Analysis of significant differences in relative abundance of bacterial community composition

| Soil depth  (cm) | Treatment | Proteobacteria | Acidobacteriota | Chloroflexi | Actinobacteriota | Gemmatimonadota | Bacteroidota | Myxococcota | Methylomirabilota | Desulfobacterota | MBNT15 | Nitrospirota |
| --- | --- | --- | --- | --- | --- | --- | --- | --- | --- | --- | --- | --- |
| 0-20 | RT | b | d | d | a | c | a | b | b | e | e | \ |
|  | IRT | c | b | b | d | a | b | a | a | c | a | \ |
|  | I70 | a | c | a | b | a | b | ab | c | d | d | \ |
|  | IS | c | c | b | c | b | b | ab | c | a | c | \ |
|  | IO | d | a | c | e | a | c | ab | a | b | b | \ |
| 20-40 | RT | a | d | c | a | e | \ | d | d | d | d | e |
|  | IRT | c | b | a | c | b | \ | b | c | b | c | c |
|  | I70 | d | a | b | d | a | \ | a | a | c | b | d |
|  | IS | c | c | a | c | d | \ | c | b | a | c | a |
|  | IO | b | b | a | b | c | \ | b | d | c | a | b |

Note: The significant differences in two soil layers over two years were represented by different lowercase letters (p < 0.05)
