# Supplementary material for: Rice-eel system combined with exogenous organic waste improves soil quality under nitrogen deficiency by regulating soil microbial community
Source: Front Microbiol. 2026 Jan 14;16:1743071. doi: 10.3389/fmicb.2025.1743071 (PMC12847270; doi:10.3389/fmicb.2025.1743071)
Supplement: Supplementary file 4 [file Table_4.DOCX]

**Supplementary table S4** Analysis of significant differences in relative abundance of fungal community composition

| Soil depth  (cm) | Treatment | Ascomycota | Basidiomycota | Chytridiomycota | Olpidiomycota | Mortierellomycota | Rozellomycota |
| --- | --- | --- | --- | --- | --- | --- | --- |
| 0-20 | RT | a | b | b | a | a | / |
|  | IRT | a | b | b | a | a | / |
|  | I70 | a | b | b | a | a | / |
|  | IS | c | b | a | a | a | / |
|  | IO | b | a | ab | a | a | / |
| 20-40 | RT | a | a | c | a | / | a |
|  | IRT | a | b | bc | a | / | a |
|  | I70 | a | b | bc | a | / | a |
|  | IS | a | b | a | a | / | a |
|  | IO | a | ab | ab | a | / | a |

Note: The significant differences in two soil layers over two years were represented by different lowercase letters (p < 0.05).
