# Supplementary material for: Rice-eel system combined with exogenous organic waste improves soil quality under nitrogen deficiency by regulating soil microbial community
Source: Front Microbiol. 2026 Jan 14;16:1743071. doi: 10.3389/fmicb.2025.1743071 (PMC12847270; doi:10.3389/fmicb.2025.1743071)
Supplement: Supplementary file 5 [file Table_5.DOCX]

**Supplementary table S5** Significance analysis of the relative abundance of the expression function of bacterial communities

| Function name | 0-20 cm | | | | |  | 20-40 cm | | | | |
| --- | --- | --- | --- | --- | --- | --- | --- | --- | --- | --- | --- |
|  | RT | IRT | I70 | IS | IO |  | RT | IRT | I70 | IS | IO |
| PWY-3781 | d | b | c | c | a |  | d | b | a | c | b |
| PWY-5101 | d | b | c | b | a |  | d | c | a | a | b |
| ILEUSYN-PWY | d | b | c | b | a |  | d | c | a | a | b |
| VALSYN-PWY | d | b | c | b | a |  | d | c | a | a | b |
| PWY-7111 | d | b | c | b | a |  | c | b | b | a | ab |
| BRANCHED-CHAIN-AA-SYN-PWY | d | b | c | b | a |  | c | b | a | a | b |
| P42-PWY | e | c | d | b | a |  | d | c | b | a | c |
| PWY-5104 | d | b | c | b | a |  | e | d | b | a | c |
| PWY-5667 | d | b | c | b | a |  | e | c | a | b | d |
| PWY0-1319 | d | b | c | b | a |  | e | c | a | b | d |
| PWY-6969 | e | b | d | c | a |  | d | c | a | b | c |
| NONOXIPENT-PWY | d | b | c | b | a |  | e | c | a | b | d |
| TCA | e | b | d | c | a |  | e | c | a | b | d |
| PHOSLIPSYN-PWY | e | c | d | b | a |  | e | c | a | b | d |
| PWY-5103 | d | b | c | b | a |  | c | b | a | a | b |
| PWY-5973 | a | c | d | c | b |  | d | c | a | b | c |
| PWY-7663 | a | c | d | c | b |  | d | c | a | b | c |
| PWY-7219 | d | b | c | b | a |  | c | b | a | a | b |
| PWY-7208 | d | b | c | b | a |  | e | c | a | b | d |
| PWY-7229 | d | b | c | b | a |  | c | b | a | a | b |

Note: The significant differences in two soil layers over two years were represented by different lowercase letters (p < 0.05).
