# Supplementary material for: Rice-eel system combined with exogenous organic waste improves soil quality under nitrogen deficiency by regulating soil microbial community
Source: Front Microbiol. 2026 Jan 14;16:1743071. doi: 10.3389/fmicb.2025.1743071 (PMC12847270; doi:10.3389/fmicb.2025.1743071)
Supplement: Supplementary file 6 [file Table_6.DOCX]

**Supplementary table S6** Significance analysis of the relative abundance of the expression function of fungal communities

| Function name | 0-20 cm | | | | |  | 20-40 cm | | | | |
| --- | --- | --- | --- | --- | --- | --- | --- | --- | --- | --- | --- |
|  | RT | IRT | I70 | IS | IO |  | RT | IRT | I70 | IS | IO |
| PWY-3781 | c | b | ab | a | a |  | d | ab | a | cd | bc |
| GLYOXYLATE-BYPASS | ab | ab | b | a | ab |  | ab | ab | a | b | a |
| PWY-5659 | d | b | c | b | a |  | b | a | a | a | b |
| PWY-7219 | a | b | b | a | b |  | a | b | a | b | b |
| PWY-7111 | a | b | b | a | b |  | a | ab | a | b | ab |
| NONOXIPENT-PWY | a | b | b | a | b |  | bc | a | bc | ab | c |
| TRNA-CHARGING-PWY | b | c | c | a | b |  | ab | b | a | b | ab |
| PWY-7229 | b | c | b | a | bc |  | ab | bc | a | c | bc |
| VALSYN-PWY | a | ab | b | a | ab |  | a | a | a | a | a |
| PWY-6126 | b | b | a | a | b |  | a | b | a | b | b |
| SER-GLYSYN-PWY | a | a | b | a | a |  | a | a | a | a | a |
| PWY-7007 | c | ab | bc | d | a |  | ab | a | c | b | c |
| PENTOSE-P-PWY | c | ab | bc | c | a |  | a | a | a | a | b |
| PWY-7228 | ab | b | a | ab | b |  | a | b | a | ab | a |
| PWY-7184 | a | a | a | a | a |  | a | b | ab | ab | ab |
| PANTO-PWY | b | b | b | a | b |  | a | b | a | b | a |
| PWY-7208 | ab | b | a | ab | ab |  | a | a | a | a | a |
| PWY-7221 | ab | c | a | b | c |  | abc | c | ab | bc | a |
| PWY-922 | b | b | b | a | b |  | ab | a | ab | b | ab |
| THRESYN-PWY | ab | bc | ab | a | c |  | ab | b | a | b | ab |

Note: The significant differences in two soil layers over two years were represented by different lowercase letters (p < 0.05).
